# Prevalence and risk factors of gross neurologic deficits in children after severe malaria: A systematic review and meta-analysis

**DOI:** 10.1371/journal.pone.0333258

**Published:** 2026-03-17

**Authors:** Allen Eva Okullo, Simple Ouma, Chandy C. John, Michael Boele van Hensbroek, Kevin Ouma Ojiambo, Caroline Otike, Alison Annet Kinengyere, Andrea L. Conroy, Moses Ocan, Ekwaro A. Obuku, Richard Idro

**Affiliations:** 1 Department of Paediatrics, Amsterdam University Medical Centers, University of Amsterdam, Amsterdam, The Netherlands; 2 Global Health Uganda, Kampala, Uganda; 3 Africa Center for Systematic Reviews & Knowledge Translation, College of Health Sciences, Makerere University, Kampala, Uganda; 4 Department of Paediatrics and Child Health, School of Medicine, College of Health Sciences, Makerere University, Kampala, Uganda; 5 Ryan White Center for Pediatric Infectious Diseases and Global Health, Indiana University School of Medicine, Indianapolis, Indiana, United States of America; 6 Clinical Epidemiology Unit, School of Medicine, College of Health Sciences, Makerere University, Kampala, Uganda; 7 Joint Clinical Research Centre, Kampala, Uganda; 8 Sir Albert Cook Library, College of Health Sciences, Makerere University, Kampala, Uganda; 9 Department of Pharmacology & Therapeutics, School of Biomedical Sciences, College of Health Sciences, Makerere University, Kampala, Uganda; 10 Faculty of Epidemiology & Population Health, London School of Hygiene & Tropical Medicine, London, United Kingdom; Federal University Dutse, NIGERIA

## Abstract

**Background:**

Children with severe malaria may develop gross neurologic deficit(s). We conducted a systematic review on the prevalence and risk factors of gross neurologic deficits after childhood severe malaria.

**Methods:**

The systematic review was conducted following PRISMA guidelines. Article search was conducted in MEDLINE, EMBASE, Web of Science, and Global Index Medicus. Studies included reported on prevalence and/or risk factors of gross neurologic deficits after severe malaria in children. Risk of bias analysis and heterogeneity assessment were performed using ROBINS tool and I^2^-statistic, respectively. Data analysis was done using quantitative synthesis in R *ver*4.5.0 software, and narrative synthesis.

**Results:**

41 studies from 16 countries in Sub-Saharan Africa and Asia comprising 11,635 children were included in the analysis. Gross neurologic deficits included motor, movement, sensory, and speech impairments. 31 studies included prevalence of gross neurologic deficits at hospital discharge (cerebral malaria (CM), n = 26, broader forms of severe malaria, n = 5). Prevalence of deficits at hospital discharge in children with CM was 15.2% (95%CI: 11.5–18.8) (I^2^ = 89.1%), compared to 2.4% (95%CI: 2.0–2.8) (I^2^ = 36.6%) in children with broader forms of severe malaria. Prevalence of deficits in CM decreased from 15.2% to 5.7% (95%CI: 1.2–10.2) (I^2^ = 79.2%) after 12 months follow-up. At regional level, Sub-Saharan Africa had a prevalence of 14.2% (95%CI: 10.5–17.9) (I^2^ = 94.9%) compared to East Asia & Pacific at 2.3% (95%CI: 0.8–3.8) (I^2^ = 0.0%) and South Asia at 6.1% (95%CI: 0.0–14.8) (I^2^ = 0.0%). Risk factors for gross neurologic deficits included profound coma, coma lasting ≥48 hours, multiple convulsions, hypoglycaemia, and acute kidney injury.

**Conclusions:**

Gross neurologic deficits are more prevalent in children in Sub-Saharan Africa with CM compared to severe malaria in general, and a number of clinical factors in children with CM increase the risk. Interventions by clinicians should target children with CM at highest risk during admission.

## Introduction

Gross neurologic deficit (GND) refers to an abnormal neurologic function of a specific body part arising from injury to the brain, spinal cord, muscles or nerves that feed the affected area [1]. This may manifest in the form of a motor deficit (spasticity, cranial nerve palsies and central hypotonia), movement disorder (ataxia, tremors and dystonia), sensory deficit (visual or hearing) or speech and language impairments (aphasia) [2]. Severe malaria, most notably cerebral malaria (CM), has been implicated as a leading cause of brain injury [3] with about 11% of children surviving CM having GND at hospital discharge [2].

Some GND in children with severe malaria may be transient, while others may be long term evidenced by a number of studies that have reported marked declines in the prevalence of GND a few to many months following hospital discharge, and persistence in a subset of individuals [4–9]. Some deficits such as cortical blindness are generally transient while others such as plegia, paresis, extrapyramidal features, and epilepsies persist [1].

A number of factors have been reported to increase the risk for neurological sequelae following CM among children. These include deep and prolonged coma, multiple seizures, recurrent hypoglycaemia, acute kidney injury, anaemia, intracranial hypertension, age younger than 3years, male sex, and a biphasic clinical course marked by recovery of consciousness followed by recurrence of convulsions and coma [3,5,10–17]. However some recent studies have not confirmed the importance of previously associated factors such as anaemia, age, sex, multiplicity of convulsions, and persistence of GND after CM [1]. In addition, no systematic review has synthesized evidence on the prevalence or risk factors of GND among children after severe malaria [1].

Given that CM is a leading cause of brain injury, knowledge on important risk factors and prevalence of GND after SM may act as a guide to policy makers and clinicians in developing and targeting interventions to those most at risk, to prevent and control these deficits. We therefore performed a systematic review and meta-analysis to synthesize evidence on the prevalence and risk factors for GND after SM in childhood.

## Methods

The systematic review and meta-analysis was done following a protocol which was registered in the International Prospective Register of Systematic Reviews (CRD42022297109) and published [1] (S1 Protocol). We conducted the review in accordance with the Preferred Reporting Items for Systematic Reviews and Meta-Analyses (PRISMA) statement [18] (S1 Checklist).

### Search strategy

An experienced librarian and information specialist (AAK) searched MEDLINE, Embase, Web of Science, and Global Index Medicus (GIM) from the earliest date to 2^nd^ February 2025 to identify studies that reported on GND after severe malaria in childhood. The search was performed using a combination of search terms, their synonyms and MeSH (Medical Subject Headings) whose details are provided in the published protocol [1] (S1 Protocol). The terms were combined using the Boolean operators ‘AND’ and ‘OR’. We searched the bibliographies of all included studies for additional eligible studies. There were no language restrictions. Google Translate was used to translate eligible articles from French to English.

### Eligibility criteria

The review included all published studies from the earliest date that reported on prevalence and/or risk factors of GND after WHO-defined severe malaria in children (persons younger than 18 years). Study designs included: cross-sectional, case-control, cohort, randomized controlled trials, quasi-experimental study, or case series. The study setting included all malaria affected regions globally. Details of the eligibility criteria are in the published protocol [1] (S1 Protocol).

### Selection process and data management

EndNote software was utilized to initially organize references obtained from the search results. Duplicate entries were removed after importing the articles into EndNote v20. Following this, the studies were screened in duplicate using predefined eligibility criteria. The review team (AEO, SO, KOO, & CO) independently screened the articles in pairs using a pre-designed and pilot-tested screening tool within EPPI-Reviewer v6.16.0.0. Any conflicts during the screening process were resolved through consensus, with a third reviewer acting as a tiebreaker when needed.

### Data abstraction

A data abstraction tool was developed in EPPI-Reviewer which included key variables for abstraction from each article, namely: study characteristics (author, year of publication, year of data collection, title, citation, institution, country, language, source of funding), study design, population characteristics (sample size, age at diagnosis of severe malaria), type of gross neurologic deficit (including author specific definitions), duration of gross neurologic deficit, type of assessment for gross neurologic deficit, risk factors, measures of association (such as risk ratios, odds ratios), and prevalence of gross neurologic deficit [1] (S1 Protocol). The tool was initially tested on 5% of the eligible studies to confirm its effectiveness in capturing all necessary information before being uploaded to EPPI-Reviewer v6.16.0.0. The four reviewers (AEO, SO, KOO, & CO) independently abstracted the data, and any disagreements were resolved through discussion. Coding was independently performed by pairs of research team members and again, any discrepancies addressed through discussion and consensus. The finalized data was then reviewed by an independent senior reviewer to verify accuracy and ensure quality control and assurance.

### Risk of bias assessment

Risk of bias assessment was carried out using ROBINS-E (‘Risk Of Bias In Non-randomized Studies- of Exposures’) tool for non-randomized controlled trials (https://www.riskofbias.info/welcome/robins-e-tool) by the four reviewers (AEO, SO, KOO, & CO) in duplicate. This is a domain-based tool and risk of bias in included studies was assessed as a judgment (low risk of bias, some concerns, high risk of bias, very high risk of bias) using seven domains. These domains are risk of bias due to confounding; risk of bias arising from the measurement of the exposure; risk of bias in the selection of participants into the study (or into the analysis); risk of bias due to post-exposure interventions; risk of bias due to missing data; risk of bias arising from the measurement of the outcome; risk of bias in the selection of the reported result.

### Data analysis and synthesis

The pooled prevalence with 95% confidence of gross neurological deficits (GND) was analysed as the outcome in this study. Effect sizes were statistically pooled using Restricted Maximum Likelihood (REML) Random Effects Meta-analysis in R version 4.5.0 [19]. REML Random Effects Meta-Analysis uses restricted (or residual) maximum likelihood, which adjusts for the degrees of freedom lost by estimating fixed effects and provides a less biased and typically more accurate estimate of heterogeneity, this was applied because heterogeneity was moderate to high. The individual studies included in this review were drawn from different populations and contexts; hence, the random effects model was used to calculate the pooled mean effect size (proportion). These effects models are usually recommended when collecting data from a series of studies where the effect size is expected to vary from one to the next, and studies are unlikely to be functionally equivalent [20], as was the case for this review, which included studies from across the globe. Furthermore, random effects models enable statistical inferences to be made to a population of studies other than those included in the meta-analysis.

The pooled effect size was transformed using Freeman–Tukey double arcsine transformation to stabilize the variance between studies and was back transformed and expressed as a raw proportion for presentation. In studies where multiple effect sizes were reported from the same sample the mean of the combined effect sizes was calculated. In cases where studies used overlapping samples, an overall estimate was calculated and those that report effect sizes from independent subgroups, each subgroup was treated as a separate sample in the meta-analysis.

The synthesis is presented in form of a summary of findings tables, simple graphs, and forest plots, as appropriate. This followed the format of the Cochrane Consumer and Communication Review Group [21]. We described the included articles, organized and tabulated the results to identify patterns and converted the results into a common descriptive format. We undertook the form of outcome data tables, simple graphs, and forest plots, as appropriate. These was incorporated into the summary of findings tables, which informed the syntheses for dissemination hence we used both narrative and quantitative synthesis. Since data on each of the risk factors reported was not adequate to pool the effect sizes, we performed a narrative synthesis without meta-analysis (SWiM) [22] using the direction of effect approach.

### Assessment of heterogeneity

The level of heterogeneity in the articles was established using I^2^ statistic. The I^2^ statistic was displayed as the percentage (%) of heterogeneity due to between-study variation [23]. The value of I^2^ statistic ≤ 25%, ≤ 50%, ≤ 75% ≥ 75% indicated low, medium, substantial and high heterogeneity among studies, respectively. Increase in heterogeneity implies underlying contextual differences between studies and limits generalizability. Subgroup analysis was performed on articles with substantial to high heterogeneity [24].

### Sub-group & sensitivity analysis

The sensitivity analysis was done by removing studies from the meta- analysis one-by-one to see if the results of the meta-analysis are sensitive to any single study. We also examined sensitivity of findings to risk of bias status (good, fair and poor quality). Additional sub-group analyses were performed to compare prevalence of GND by: duration of follow-up, that is, at discharge, three, six, 12 and over 12-months follow-up; by region, that is, Sub-Saharan Africa, East Asia & Pacific, and South Asia region, since studies were found to report on GNDs at different follow-up time-points and in different regions; by study design; and by type of treatment.

### Publication bias

We assessed the risk of publication bias in the included articles by using the asymmetry of funnel plots [25]. This technique uses ranking methods for data augmentation and has demonstrated reliability in evaluating publication bias caused by missing studies or data. A funnel plot was employed to visually examine the symmetry of effect sizes, aiding in the detection of potential bias, particularly when smaller studies tend to report more pronounced effects. In the absence of missing studies or small study effects, the scatter plot looks like a symmetrical inverted funnel with a wide base and a narrow top [25]. The presence of large “holes” or asymmetry in the plot suggests publication bias, however this might also be explained by other factors such as study heterogeneity.

### Dealing with missing data

In case of missing data from the published articles, study authors were contacted. When the author could not be accessed or in case of no response from authors, we reported the characteristics of the study but did not include such a study in the meta-analysis.

### Deviations from the protocol

The effect size used was a raw proportion of children who developed GNDs as opposed to the Freeman-Tukey double arcsine transformation approach as stated in the protocol. We used the random effects model because this method accounts for heterogeneity between studies, which is expected in this study due to variations in demographics and study design, and it assumes that the true effect could vary from study to study [26]. While the fixed effects model assumes that one true effect size underlies all studies in a meta-analysis and that any differences are due to sampling error [26].

We compared the prevalence of GND among children in studies specifically enrolling children with CM compared to studies with broader forms of SM (including CM), which is a deviation from the protocol that indicated that we would compare the prevalence of GND in studies with CM compared to studies with non-CM. This was because studies that reported GND in different forms of SM did not disaggregate them by SM criteria, therefore we could not determine the prevalence of GND in the non-CM group.

We did not perform a meta-analysis for different age-groups as stated in the protocol because most of the studies did not disaggregate prevalence of GND by age-group and there were variations in the age groups reported.

## Results

### Study selection

**Fig 1:**
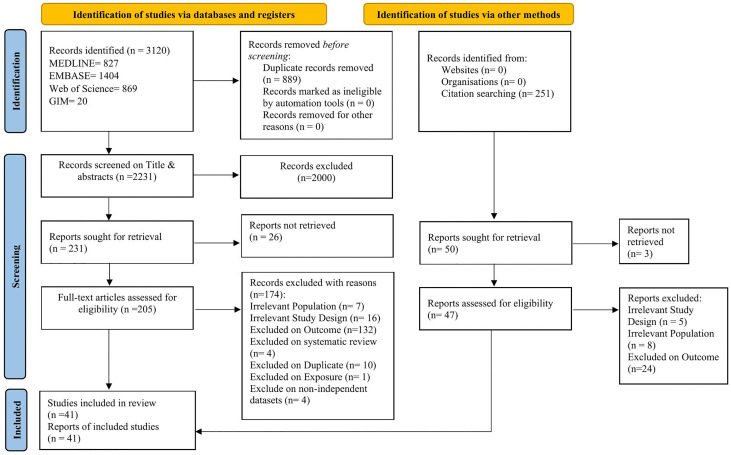
PRISMA flow chart showing the study selection process.

An initial search of databases namely, MEDLINE, Embase, Web of Science, and Global Index Medicus from 1946 up to 11 June 2023, yielded 2856 potentially relevant publications (MEDLINE = 750, EMBASE = 1249, Web of Science = 847, GIM = 10). An updated search was conducted up to 2^nd^ February, 2025, which yielded a total of 264 potentially relevant publications (MEDLINE = 77, EMBASE = 155, Web of Science = 22, GIM = 10). Of the total records identified from both searches (3120), 889 were excluded after duplicate removal, resulting in 2231 records for title and abstract screening (Fig 1). A total of 2000 records were excluded after title and abstract screening, resulting in 231 articles. Full-texts of twenty-six articles were not retrieved, resulting in 205 articles for full-text screening. After full-text screening, 174 did not meet the eligibility criteria and were excluded. An additional 10 studies that met the eligibility criteria were identified through searching the bibliography of the included studies. A total of forty-one studies met the eligibility criteria and were included in the systematic review (Fig 1).

### Characteristics of included studies

Among the forty-one studies, thirty-four reported on GND after cerebral malaria (CM), while seven reported on GND after different forms of severe malaria. These studies were from 16 countries in three regions, namely: i) Sub-Saharan Africa (Uganda, The Gambia, Kenya, Nigeria, Malawi, Togo, Mozambique, Ghana, Tanzania, Rwanda, Democratic Republic of Congo, South Sudan, Mali); ii) East Asia & Pacific (Papua New Guinea); and iii) South Asia (India, Pakistan) (Table 1). The types of GND included: motor deficits such as paresis, plegia, hypertonia, hypotonia, dystonia, divergent squint, oral dyspraxia; movement deficits such as ataxia; sensory deficits described as deafness, impaired hearing, cortical blindness, visual impairment; speech impairments described as delayed speech, and dysphasia; and a vegetative state. Thirty-one studies reported GND at discharge, two studies reported GND at three months, seven studies reported GND at six months, three studies reported GND at 12-months, and five studies reported GND after 12 months. Nine studies reported adjusted odds ratios (AOR) for the association between risk factors and GND (Table 2). Study designs included, prospective cohort (n = 32), retrospective cohort (n = 3), Randomized-Controlled Trials (n = 4), and cross-sectional (n = 2). Among studies that reported on treatment, types of treatment included: chloroquine (IV or subcutaneous) (n = 2), chloroquine or IV quinine (n = 2), IV quinine (n = 6), ACTs (IV artesunate or IM Artemether) or IV quinine (n = 8) and ACTs (n = 4). The review had a total of 11,635 children with ages ranging from one month to 15yrs. A list of the excluded studies at full text along with the reasons for exclusion has been provided as an additional file, S1 Table.

**Table 1:** Summary of included studies on prevalence of gross neurologic deficits after childhood severe malaria.

Study (Year)	Country	Type of SM	Study Design	Type of Gross Neurologic Deficit	Prevalence at Discharge n/N (%)	Prevalence after discharge n/N (%)
Schmutzhard et al (1984) [27]	Tanzania	CM	Prospective cohort	Hemiparesis	NR	4/49 (8.2%) at 6 months
Ahmad et al (1986) [28]	India	CM	Prospective cohort	Hemiparesis, monoparesis, isolated cranial nerve palsy (ptosis and facial)	1/20 (5%)	
Molyneux et al (1989) [4]	Malawi	CM	Prospective cohort	Hemiparesis, generalized hypotonia, generalized spasticity of limbs, cerebellar ataxia, bilateral 3–4/s extrapyramidal tremor	12/111 (10.8%)	7/111 (6.3%) at 1 month
Brewster et al (1990) [5]	The Gambia	CM	Prospective cohort	Hemiplegia, cortical blindness, aphasia, ataxia, generalised spasticity, tremors	32/265 (12.1%)	6/265 (2.3%) at 6 months
Bondi et al (1992) [10]	Nigeria	CM	Prospective cohort	Monoparesis, cortical blindness, hearing loss, dysphasia, dysarthria, spastic quadriplegia, hemiparesis, hemiplegia	7/62 (11.3%)	
Schapira et al (1993) [29]	Mozambique	CM	Randomized Controlled Trial	Cortical blindness, inferior paraparesis	4/54 (4.4%)	
Walker et al (1993) [30]	Nigeria	CM	Randomized Controlled Trial	Motor weakness, cortical blindness, loss of neurological milestones	5/45 (11.1%)	
Steele et al (1995) [31]	Ghana	CM	Retrospective cohort	Cortical blindness, decreased vision, hypotonia, spastic hemiplegia, deafness, aphasia, gait disturbance, hemiplegia, facial palsy	42/187 (22%)	
Newton et al (1996) [32]	Kenya	CM	Prospective cohort	Hemiparesis, blindness, hand dystonia, divergent squint, mild diplegia, vegetative state	11/44 (25%)	
Crawley et al (1996) [33]	Kenya	CM	Prospective cohort	Hemiplegia, spastic quadriplegia	NR	7/58 (12%) at 3 months
Allen et al (1996) [34]	Papua New Guinea	CM	Prospective cohort	Severe spastic quadriparesis, severe spastic quadriplegia, poor co-ordination of the left arm and hand, mild spasticity in both legs	NR	4/66 (6%) at 6 months
Genton et al (1997) [35]	Papua New Guinea	CM	Prospective cohort	Right hemiplegia, involuntary movements	2/118 (1.7%)	2/50 (4%) at 1 month
Van Hensbroek et al (1997) [11]	The Gambia	CM	Prospective cohort	Paresis, ataxia, hearing defects, visual field defects, aphasia, developmental regression	NR	29/466 (6.2%) at 1 month, 18/452 (4.0%) at 6 months
Assimadi et al (1998) [36]	Togo	SM	Prospective cohort	Aphasia, hemiplegia, oculomotor paralysis, cerebellar ataxia	9/445 (2%)	
Olumese et al (1999) [6]	Nigeria	CM	Prospective cohort	Cortical deafness, abnormal gait, aphasia, right hemiparesis, severe hypotonia and other motor deficits	12/103 (11.7%)	1/103 (1%) at 1 month
Crawley et al (2001) [37]	Kenya	CM	Prospective cohort	Hemiplegia, spastic quadriplegia	6/58 (10.3%)	
Oguche et al (2002) [7]	Nigeria	CM	Prospective cohort	Spastic quadriparesis/aphasia, motor deficits/regression of motor milestone, hearing impairment/abnormal behavior	5/39 (12.8%)	1/39 (2.6%) at 1 month
Idro et al (2004) [12]	Uganda	CM	Prospective cohort	Cortical blindness, impaired hearing, spastic quadriparesis, aphasia, generalised hypotonia	3/93 (3.2%)	
Idro et al (2005) [38]	Kenya	CM	Cross-sectional	Motor impairments (central hypotonia, paresis, ataxia), aphasia, blindness, deafness	39/354 (11%)	
Idro et al (2006) [3]	Kenya	CM	Prospective cohort	Motor impairments, speech & language impairments, hearing & visual impairment	NR	22/143 (15.4%) median 64 months (IQR 40–78)
Ngoungou et al (2006) [39]	Mali	CM	Prospective cohort	Speech delay, oral dyspraxia, diplegia, dystonia	NR	5/101 (5%) at about 36 months
Medana et al (2007) [40]	Kenya	CM, PR, MS	Prospective cohort	Quadriparesis, generalized hypotonia	3/117 (2.6%)	
Ahmed et al (2008) [41]	Pakistan	CM	Retrospective cohort	Motor deficit, cranial nerve palsy	1/9 (11.0%)	
Casals-Pascual et al (2008) [42]	Kenya	CM	Retrospective cohort	Visual impairment, impairment of speech, motor impairment (hemiparesis, quadriparesis, monoparesis)	32/108 (30%)	
Opoka et al (2009) [8]	Uganda	CM	Prospective cohort	Hyperreflexia and hypertonia, spastic quadriplegia, vision and hearing impairments, ataxia, lack of coordination and attention deficit	19/76 (25.0%)	7/76 (9.2%) at 3 months, 2/74 (2.7%) at 6 months, 1/68 (1.5%) at 24 months
Birbeck et al (2010) [13]	Malawi	CM	Prospective cohort	Hemiparesis, monoparesis, hypotonia, gait ataxia, speech regression, choreiform movements, spastic quadriparesis, language regression, cortical blindness	12/132 (13.6%)	
Dondorp et al (2010) [43]	Mozambique, The Gambia, Ghana, Kenya, Tanzania, Nigeria, Uganda, Rwanda, D.R.C	SM	Randomized Controlled Trial	Severe motor impairment, cortical blindness, severe speech and hearing impairment	115/4898 (2.3%)	191/507 (37.7%) at 28 days~1 month
Bhanushali et al (2011) [44]	Malawi	CM	Prospective cohort	Motor deficits, sensory deficits, ataxia	NR	4/25 (16%) at 12 months
Manning et al (2011) [45]	Papua New Guinea	CM, SMA, RD, MS, PR	Prospective cohort	Cortical blindness, motor deficits (ranging from mild ataxia to spastic quadriparesis)	7/261 (2.7%)	
Oluwayemi et al (2013a) [46]	Nigeria	CM	Prospective cohort	Ataxia, speech impairment, cortical blindness, hearing impairment, hemiparesis, spastic quadriplegia, spastic triplegia, quadriparesis, left-sided ptosis	22/57 (38.6%)	
Oluwayemi et al (2013b) [47]	Nigeria	CM	Prospective cohort	Visual impairment, speech impairment, monoparesis, quadriparesis, hearing impairment, movement disorder	16/131 (12%)	
Mergani et al (2015) [48]	South Sudan	CM	Cross-sectional	Hemiparesis, blindness, hemiplegia, aphasia, quadriplegia, quadriparesis	64/351 (18%)	
Hawkes et al (2015) [49]	Uganda	All SM	Randomized controlled trial	Inability to sit, spastic or flaccid paresis of one or more limbs, seizures, unilateral weakness, vision loss, gaze palsy, and poor head control	12/164 (7.3%)	
Oninia et al (2015) [50]	Nigeria	CM	Retrospective cohort	Motor deficit, cortical blindness, aphasia, hearing impairment	6/17 (35.3%)	
O’Brien et al (2018) [51]	Democratic Republic of Congo	CM	Prospective cohort	Aphasia, ataxia, central hypotonia, choreiform movements, hemiparesis, blindness	14/121 (11.6%)	14/121 (11.6%) at 1 month
Conroy et al (2021) [52]	Uganda	CM	Prospective cohort	Motor deficits, ataxia, movement disorders, speech disorders, visual disorders	83/232 (35.8%)	11/223 (4.9%) at 6 months, 7/223 (3.1%) at 12 months, 6/220 (2.7%) at 24 months
Alabi et al (2023) [14]	Nigeria	CM	Prospective cohort	Abnormal posturing, absent cornea reflexes, abnormal pupillary light reflexes, hypotonia, hypertonia, depressed tendon reflexes, hyper-reflexia	11/41 (26.8%)	
Chastang et al (2023) [15]	Malawi	CM	Prospective cohort	Abnormal hearing, movement, vision, tone, development	152/1395 (10.9%)	
Prasad (2023) et al [53]	India	CM	Prospective cohort	Motor deficit, visual impairment		7/35 (20%) at 6 months

**Table 2:** Summary of included studies on risk factors of gross neurologic deficits after childhood severe malaria.

Study (Year)	Country	Type of SM	Duration of follow-up	Risk Factors for Gross Neurologic Deficits	OR (95% CI)	AOR (95% CI)
Van Hensbroek et al (1997) [11]	The Gambia	CM	6 months	Coma score 0 or 1 Duration of coma>=48hrs Multiple convulsions	6.9 (1.9-25.3) 11.2 (3.0-42.4) 6.0 (2.0-18.3)	7.4 (1.8-29.7) 13.4 (3.4-52.4) 7.1 (2.2-22.7)
Oguche et al (2002) [7]	Nigeria	CM	1 month	Metabolic acidosis Elevated plasma creatinine	NR NR	4.5 CI NR 10.5 CI NR
Idro et al (2004) [12]	Uganda	CM	Hospital admission	Multiple convulsions	NR	12.8 (3.0–211)
Idro et al (2006) [3]	Kenya	CM	64 months	Previous admissions Previous history of seizures Age < 3 years Hypoglycemia at or during admission Features of raised intracranial pressure Multiple (3 or more seizures) Focal neurologic signs Deep coma Severe malnutrition	NR NR NR NR NR NR NR NR NR	4.8 (1.3-17.6) 7.6 (1.7-33.6) 6.4 (1.2-36) 6.1 (1.4-25.7) 6.0 (1.4-30.6) 8.3 (2.3-29.5) 12.3 (1.4-110) 28.8 (3.0-280) 6.6 (1.4-30.6)
Casals-Pascual et al (2008) [42]	Kenya	CM	Hospital admission	Admission with profound coma Convulsions	7.7 (2.5-24.0) 3.4 (1.1-10.5)	5.5 (1.5-20.7) 16.4 (2.9-90.8)
Mergani et al (2015) [48]	South Sudan	CM	Hospital admission	Abnormal posture either decerebration or decortication Focal convulsions Coma >48 hours	6.1 (3.3-11.0) 0.6 (3.6-30.7) 4.1 (2.1-8.0)	5.3 (2.7-10.6) 11.3 (5.5-23.2) 3.8 (1.9-7.9)
Conroy et al (2019) [17]	Uganda	CM & SMA	24 months	Acute kidney injury	NR	3.0 (1.2-7.6)
Namazzi et al (2022) [16]	Uganda	CM, SMA, MS, PR, RDS	Hospital admission	Acute kidney injury Unresolved acute kidney injury Elevated BUN (>20mg/dL)	2.4 (1.5-4.0) 4.6 (2.4-8.7) 2.8 (1.7-4.8)	2.0 (1.2-3.4) 3.7 (1.9-7.2) 2.8 (1.7-4.9)
Chastang et al (2023) [15]	Malawi	CM	Hospital admission	Hypoglycemia at admission	2.7	3.2 (1.5-6.9)

### Risk of bias in the included studies

Eighteen of the included studies (18/41: 43.9%) had low risk of bias, seventeen (17/41: 41.5%) had a moderate risk of bias while six (6/41: 14.6%) had high risk of bias (S1 Fig). Following the risk of bias assessment criteria used, included studies had potential risk of: bias due to confounding (12/41: 29.3%), bias due to post-exposure interventions (9/41: 22%), bias due to missing data (3/41: 7.3%), bias arising from measurement of the outcome (1/41: 2.4%), and bias in selection of the reported result (12/41: 29.3%) (S1 and S2 Figs).

### Publication bias

We used the funnel plot and Egger’s test to assess for publication bias. The funnel plot was asymmetrical which indicated presence of publication bias (S3 Fig). The Egger’s test was statistically significant (p < 0.0001) which strongly suggested evidence of small-study effects an indication of publication bias as shown in the funnel plot. The bias estimate 4.05 (Standard Error = 0.64) represented the intercept of the regression line used in Egger’s test. A value far from zero (and statistically significant) which implied that the funnel plot is asymmetric. This positive value of the bias estimate suggested that smaller studies are more likely to report higher proportions of gross neurological deficits, which may indicate that studies with low prevalence rates may be underreported or unpublished.

### Prevalence of gross neurologic deficits in children with severe malaria at hospital discharge globally

**Fig 2:**
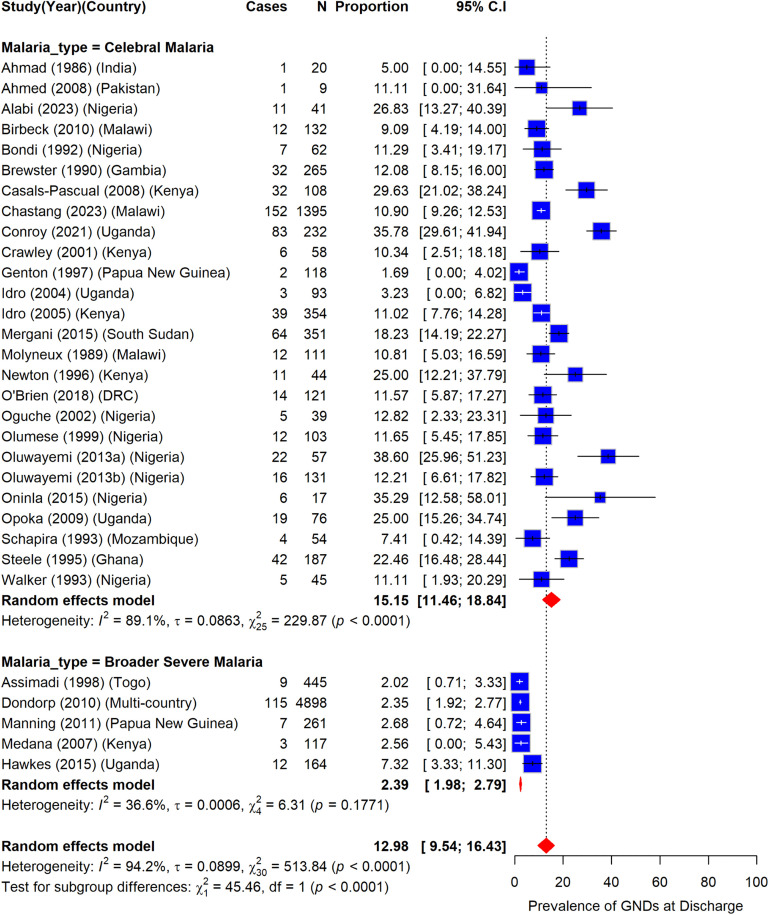
Prevalence of gross neurologic deficits among children with severe malaria at hospital discharge.

A total of 10,108 participants were included from 31 different studies to determine the prevalence of GND at hospital discharge (Fig 2). Of these, 26 studies reported GND after CM while five reported GND after different forms of SM (including CM). Among participants who only had CM, the prevalence of GND was 15.2% (95%CI: 11.5–18.8) (I^2^ = 89.1%) while among those who had broader forms of SM (including CM), the prevalence of GND was 2.4% (95%CI: 2.0–2.8) (I^2^ = 36.6%) (Fig 2). Overall, the prevalence of participants with GND after severe malaria at hospital discharge was 13.0% (95%CI: 9.5–16.4), however there was a high heterogeneity in the included studies (I^2^ = 94.2%) between the two sub-groups (Fig 2).

### Sensitivity analysis

By removing studies one by one and restricting the analysis among studies with low and moderate risk of bias (RoB), the pooled prevalence of GND among children with CM was 12.9% (95%CI:9.0–16.8) (I^2^ = 94.7%) (S4 Fig) which was not statistically different from the overall pooled prevalence of GND among children with CM at 13% (95%CI: 9.5–16.4) (I^2^ = 94.2%) (S5 Fig).

### Prevalence of gross neurologic deficits among children with cerebral malaria at 3, 6, 12 and >12 months follow-up globally

**Fig 3:**
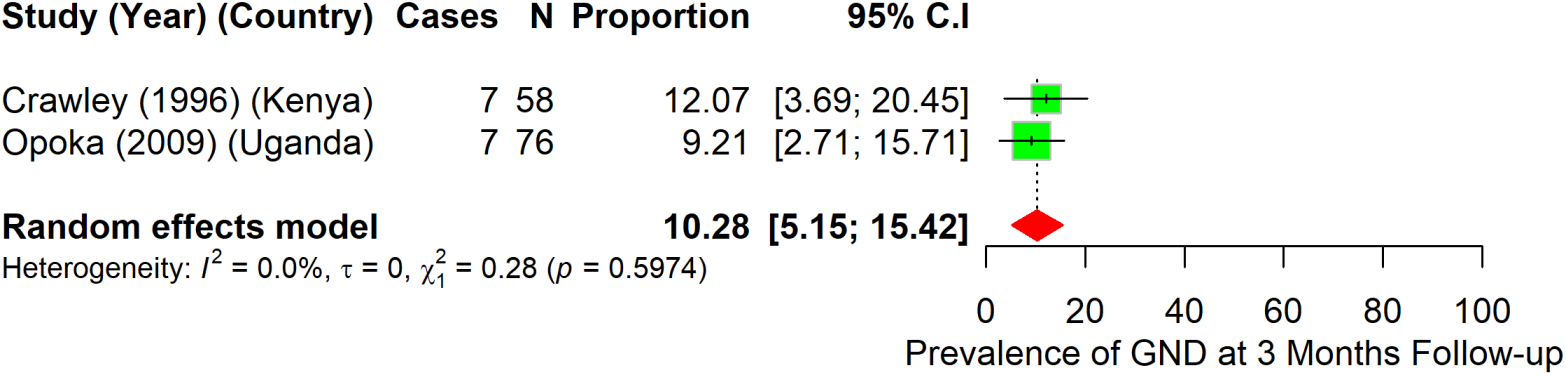
Prevalence of gross neurologic deficits among children who had CM at 3-month follow-up.

**Fig 4:**
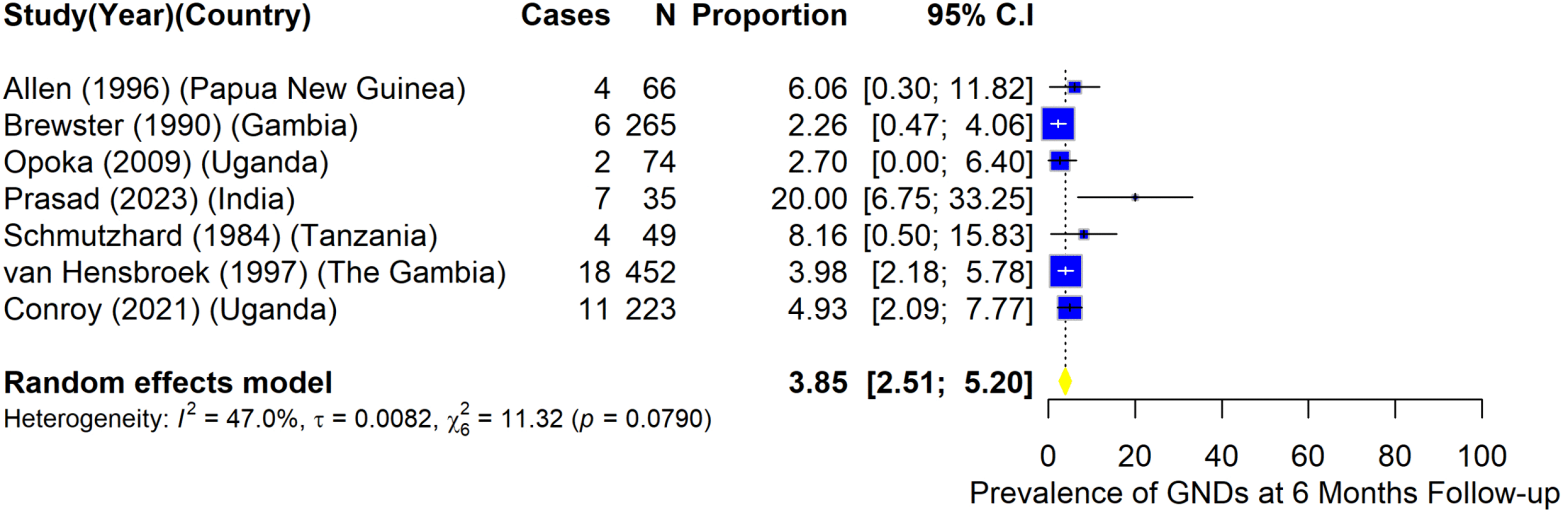
Prevalence of gross neurologic deficits among children who had CM at 6-month follow-up.

**Fig 5:**
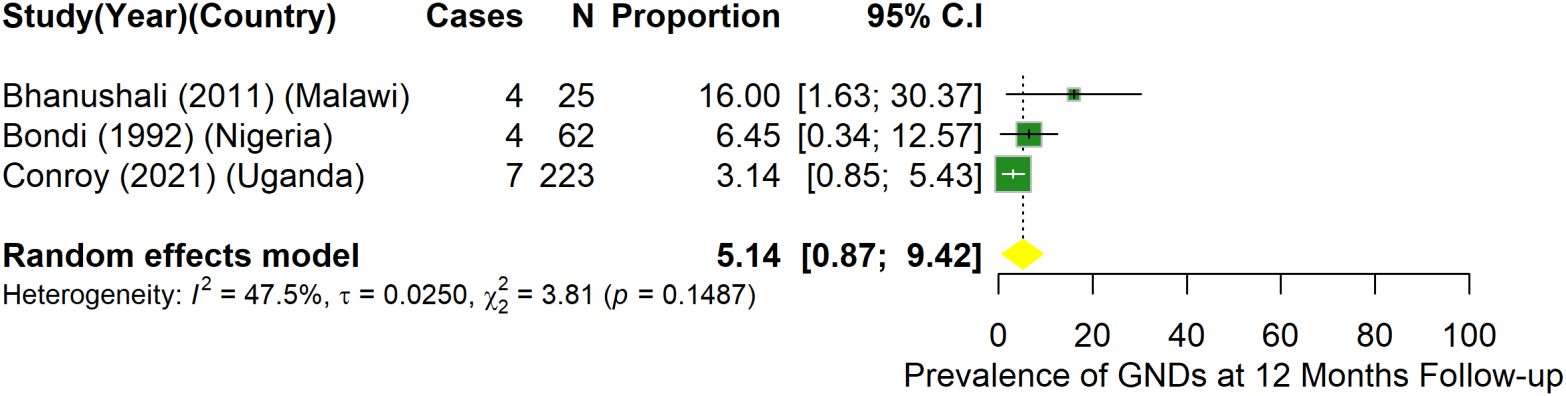
Prevalence of gross neurologic deficits among children who had CM at 12-month follow-up.

**Fig 6:**
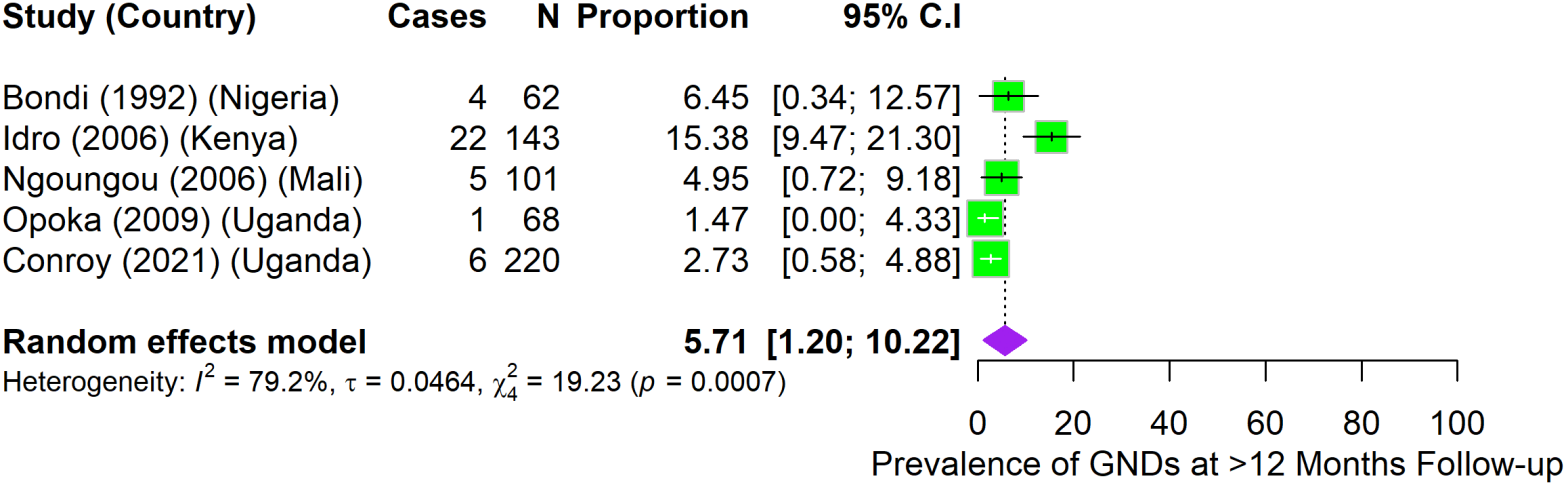
Prevalence of gross neurologic deficits among children who had CM at over 12-month follow-up.

A total of 12 studies provided data for prevalence of GND after hospital discharge (Figs 3–6).

At three months follow-up, the prevalence of GND among 134 children with CM was 10.3% (95%CI: 5.2–15.4), (I^2^ = 0.0%) (Fig 3).

Prevalence of post-discharge GND among children with CM was 3.9% (95%CI: 2.5–5.2) among studies that reported GND at six months follow-up (I^2^ = 47.0%) (Fig 4).

At 12 and over 12 months follow-up, prevalence of GND among children with CM was not statistically different at 5.1% (95%CI: 0.9–9.4) (I^2^ = 47.5%) (Fig 5), and 5.7% (95%CI: 1.2–10.2) (I^2^ = 79.2%) (Fig 6) respectively among studies that reported GND in these periods.

### Prevalence of gross neurologic deficits among children with severe malaria by region

**Fig 7:**
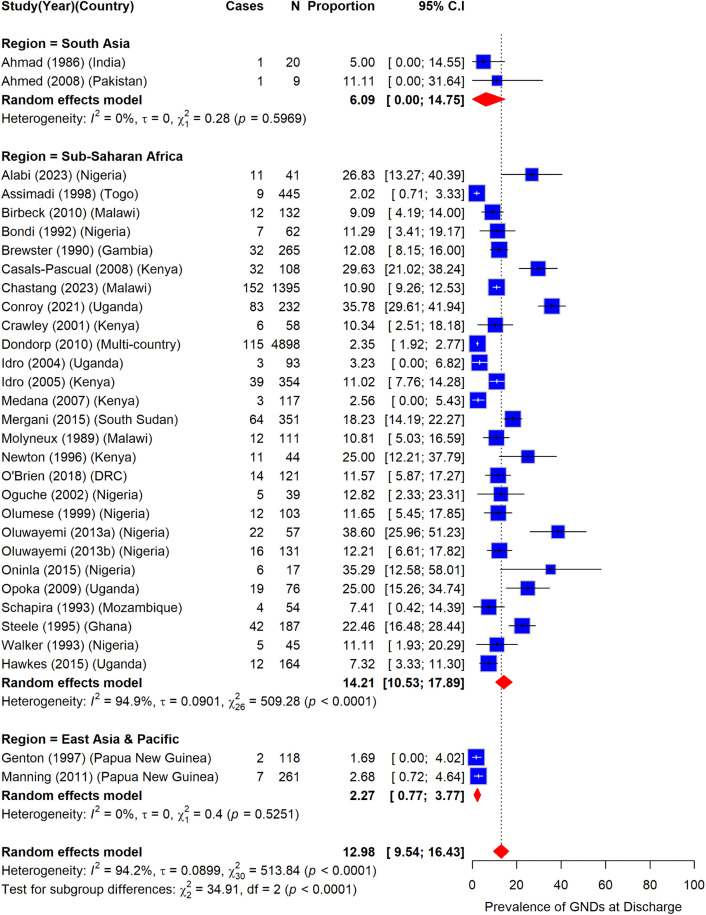
Prevalence of gross neurologic deficits among children with severe malaria by region.

Sub-Saharan Africa accounted for a significantly higher prevalence of GND among participants with severe malaria at hospital discharge at 14.2% (95%CI: 10.5–17.9) (I^2^ = 94.9%) compared to South Asia 6.1% (95%CI: 0.0–14.8) (I^2^ = 0.0%) and East Asia & Pacific, 2.3% (95%CI: 0.8–3.8) (I^2^ = 0.0%) (Fig 7).

### Prevalence of gross neurologic deficits among children with severe malaria by study design

**Fig 8:**
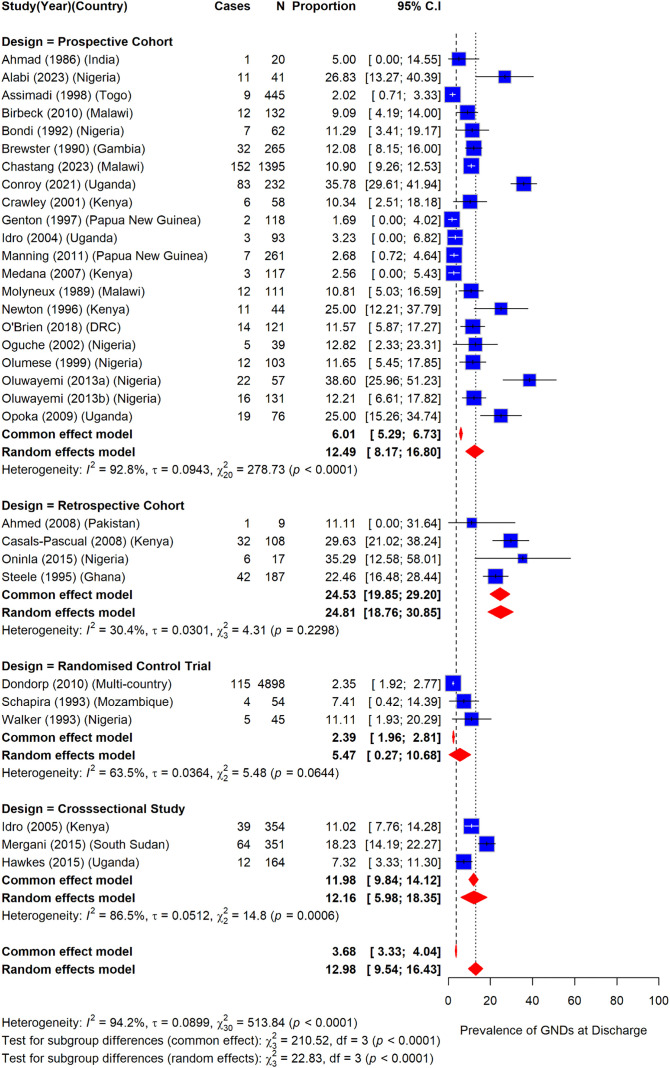
Prevalence of gross neurologic deficits among children with severe malaria by study design.

Retrospective cohort study designs reported the highest prevalence of GND among study designs at hospital discharge at 24.8% (95%CI: 18.8–30.9) (I^2^ = 30.4%) while randomized controlled trials reported the lowest prevalence at 5.5% (95%CI: 0.3–10.7) (I^2^ = 63.5%). The remaining study designs, that is, the prospective cohort and cross-sectional studies had similar prevalences at 12.5% (95%CI: 8.2–16.8) (I^2^ = 92.8%) and 12.2% (95%CI: 6.0–18.4) (I^2^ = 86.5%) (Fig 8).

### Prevalence of gross neurologic deficits among children with severe malaria by type of treatment

**Fig 9:**
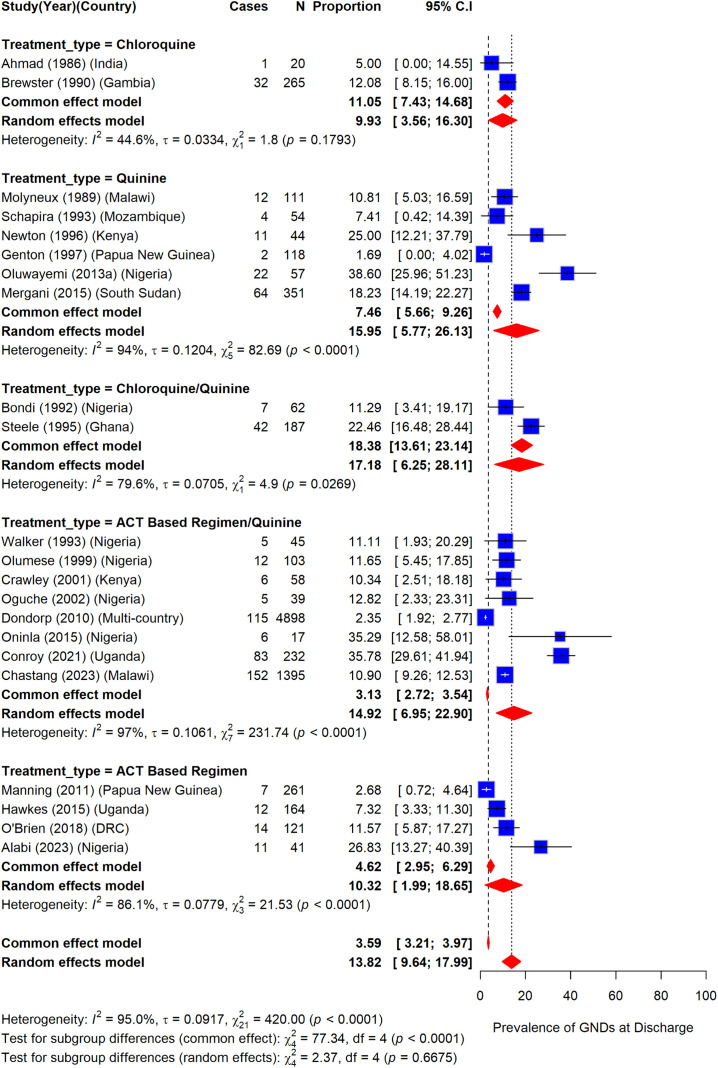
Prevalence of gross neurologic deficits at hospital discharge by treatment type.

The prevalence of GNDs among children in different treatment groups were as follows: chloroquine, 9.9% (95%CI: 3.6–16.3) (I^2^ = 44.6%); quinine, 16.0% (95%CI: 5.8–26.1) (I^2^ = 94.0%); chloroquine/quinine, 17.2% (95%CI: 6.3–28.1) (I^2^ = 79.6%); ACTs/quinine, 14.9% (95%CI: 7.0–22.9) (I^2^ = 97%); and ACTs, 10.3% (95%CI: 2.0–18.7) (I^2^ = 86.1%). However, the difference in prevalence of GNDs by treatment type was not statistically significant (P = 0.67) (I^2^ = 95%) (Fig 9).

### Risk factors for gross neurologic deficits in children with cerebral malaria globally

Nine studies reported adjusted odds ratios (AORs) for risk factors of GND following childhood cerebral malaria [3,7,11,12,15–17,42,48] (Table 2). Risk factors associated with GND at hospital discharge included: focal convulsion, AOR = 11.3, 95%CI: 5.5–23.2 [48]; unresolved acute kidney injury (AOR = 3.7, 95%CI: 1.9–7.2) along with elevated blood urea nitrogen (BUN) (>20mg/dL) (AOR = 2.8, 95%CI: 1.7–4.9) [16]; metabolic acidosis, AOR = 4.5, and elevated plasma creatinine, AOR = 10.5 [7], suggestive of acute kidney injury, which were associated with short-term deficits at 1 month. Some risk factors were associated with GND both at hospital discharge, and long-term (GND persisting >3months duration of follow-up). These included: admission with profound/deep coma, AOR = 5.5, 95%CI: 1.5–20.7 [42], AOR = 7.4, 95%CI: 1.8–29.7 [11] and AOR = 28.8, 95%CI: 3.0–280 [3]; coma lasting for ≥48hours, AOR = 13.4, 95%CI: 3.4–52.4 [11] and AOR = 3.8, 95%CI: 1.9–7.9 [48]; multiple convulsions, AOR = 7.1, 95%CI: 2.2–22.7 [11], AOR = 12.8, 95%CI: 3.0–211 [12], and AOR = 16.4, 95%CI: 2.9–90.8 [42]; hypoglycemia, AOR = 6.1, 95%CI: 1.4–25.7 [3] and AOR = 3.2, 95%CI: 1.5–6.9 [15] and acute kidney injury, AOR = 3.0, 95%CI: 1.2–7.6 [17] and AOR = 2.0, 95%CI:1.2–3.4 [16] as shown in Table 2. Risk factors associated with long-term GND included: previous admissions (AOR = 4.8, 95%CI: 1.3–17.6), focal neurological signs observed during admission (AOR = 12.3, 95%CI: 1.4–110), multiple seizures (AOR = 8.3, 95%CI: 2.3–29.5), previous history of seizures (AOR = 7.6, 95%CI: 1.7–33.6), age below 3years (AOR = 6.4, 95%CI: 1.2–36), severe malnutrition (AOR = 6.6, 95%CI: 1.4–30.6), and features of raised intracranial pressure (AOR = 6.0, 95%CI: 1.4–30.6)) [3].

## Discussion

The review shows that prevalence of GND at hospital discharge was higher in studies where children had CM compared to those where children had different forms of SM, including but not limited to CM. The prevalence of GND among children with CM decreased significantly overtime following hospital discharge. Sub-Saharan Africa had a significantly higher prevalence of GND compared to the two Asia regions and a number of clinical factors that increase the risk of GND among children with CM were reported across several studies.

Prevalence of GND at hospital-discharge in studies reporting children with CM was seven times higher than that in studies with different forms of SM. All studies reviewed reported marked declines in the prevalence of GND among children with CM within a few months post-hospital discharge [4–9,54], demonstrating that some deficits are transient and may be a marker of severe illness that resolves overtime. The prevalence was highest at hospital discharge and reduced over follow-up. At three months post discharge, the pooled prevalence of GND reduced by over 30%. There was a further decline to six months after which the prevalence remained more-or-less stable beyond 12 months follow-up. This indicates that most recovery from GND after CM occurs within the first six months after acute illness [54] after which it is less likely to resolve.

Cerebral malaria is the most severe neurological complication of severe malaria [2], which has been associated with both mortality and neurological sequelae among children [2,14,54]. While malaria with multiple seizures has been associated with GND, the burden is much lower compared to children with CM [55]. Studies which had different forms of SM were predominantly populated by other forms of SM (such as SMA and malaria with multiple seizures) which could explain the significantly lower pooled prevalence of GND among children in this group compared to those who only had CM.

Factors such as admission with profound coma, coma lasting ≥48hours, hypoglycemia, features of raised intracranial pressure, severe malnutrition, metabolic acidosis, elevated plasma creatinine, acute kidney injury, unresolved acute kidney injury along with elevated blood urea nitrogen, convulsions, multiple seizures, previous history of seizures, focal neurological signs observed during admission, previous admissions, and age below 3years were reported to increase the risk of GND after CM. The pathophysiology of CM involves the sequestration of red blood cells with parasitaemia in cerebral blood vessels which results in microvascular congestion, breakdown of the blood-brain barrier, and elevated intracranial pressure [56] which reduces cerebral perfusion pressure, nutrient and oxygen delivery [2] leading to herniation and obstruction of the blood vessels [56]. These processes may result in metabolic acidosis and elevated plasma creatinine as a result of acute kidney injury [17], and hypoglycaemia which may result in coma all of which have been implicated in neurologic sequelae [2]. It is likely that the more intense these processes leading to the coma, the deeper and more prolonged the coma, resulting into greater neurologic damage. Convulsions and seizures which may be attributed to excitation by quinolinic acid, buildup of parasitaemia as is common in childhood cerebral malaria and the resulting neuronal injury [2]. It is postulated that brain injury may be caused by the seizure initiating excitotoxic and other factors that may lead to a vicious cycle of neural injury and more seizures [2]. The reported age below 3 years as a risk factor for GND is consistent with a study in which the highest risk for motor, language and social development problems was among children under 5years [57] which suggests that GND and cognitive and social outcomes may both be worse in children with CM under 5years of age compared to older children [57].

The prevalence of GND in children with CM in Sub-Saharan Africa at hospital discharge was much higher than that in East Asia & Pacific, and South Asia regions. In a study conducted in Papua New Guinea in which the prevalence of GND among children with CM was particularly very low (1.7%), the proportion of children with hypoglycemia and deep coma, which is a marker of greater neurologic damage and a risk factor for GND [35], was notably much lower than that found in Sub-Saharan African countries [35]. A number of studies in Sub-Saharan Africa that have reported higher prevalences in GND among children with CM compared to that in reported in the East Asia & Pacific region, have also reported higher proportions of children exposed to these risk factors [5,10,11,42]. Thus, implying a correlation between the prevalence of GND and the proportion exposed to risk factors for GND, a likely reason for the comparatively higher prevalence of GND among children with CM in Sub-Saharan Africa compared to that in the East Asia & Pacific, and South Asia regions.

This review had some limitations. A number of studies did not indicate the type of examination that was used to assess GND, we therefore made an assumption that the standard neurologic examination was used which could have led to some reporting bias and might have had an effect on the outcome in the primary studies. Most of the studies did not report whether the examiners for neurologic sequelae were blinded to the exposure status of the study participants, we therefore cannot assess whether there could have been reporting bias which could have affected the outcomes reported in the primary studies. We limited the impact of this bias by conducting a risk of bias assessment and carrying out a meta-analysis on the prevalence of gross neurologic deficits among children with CM at discharge in studies with low and moderate risk of bias. The strength of this review is that it has addressed all the set objectives. We synthesized evidence on the prevalence of GND in children with severe malaria globally (in Sub-Saharan Africa, East Asia & Pacific, and South Asia), and over different periods of follow-up. We also synthesized evidence on the risk factors of GND after SM in childhood.

## Conclusions

One in every six children with CM has a GND at hospital discharge compared to one in every 50 children with different forms of SM. The burden of GND is also significantly higher in Sub-Saharan Africa compared to other regions, namely, South Asia, and East Asia & Pacific region, hence interventions for the prevention and rehabilitation of GND should target children with CM in Sub-Saharan Africa. Most GND at hospital discharge are likely markers of acute illness and are transient in nature. A number of clinical factors associated with the pathophysiology of CM increase the risk of acute and chronic GND. This calls for close monitoring of children with CM by clinicians, during admission and targeting treatment protocols suited to each child.

## Supporting information

S1 Protocol Prevalence and risk factors of gross neurologic deficits in children after severe malaria: a systematic review protocol. (PDF)

S1 Checklist PRISMA 2020 checklist. (DOCX)

S1 Table List of excluded studies on full text. (DOCX)

S1 Fig ROBINS-E Traffic plot. (TIF)

S2 Fig ROBINS-E Summary plot. (TIF)

S3 Fig Funnel plot. (TIF)

S4 Fig Prevalence of gross neurologic deficits among children with Severe malaria at hospital discharge in studies with low and moderate risk of bias. (TIF)

S5 Fig Prevalence of gross neurologic deficits among children with Severe malaria at hospital discharge by risk of bias. (TIF)

## References

[1] Okullo AE, John CC, Idro R, Conroy AL, Kinengyere AA, Ojiambo KO, et al. Prevalence and risk factors of gross neurologic deficits in children after severe malaria: a systematic review protocol. Syst Rev. 2025;14(1):76. doi: 10.1186/s13643-025-02785-4 40181467 PMC11967140

[2] Idro R, Marsh K, John CC, Newton CRJ. Cerebral malaria: mechanisms of brain injury and strategies for improved neurocognitive outcome. Pediatr Res. 2010;68(4):267–74. doi: 10.1203/PDR.0b013e3181eee738 20606600 PMC3056312

[3] Idro R, Carter JA, Fegan G, Neville BGR, Newton CRJC. Risk factors for persisting neurological and cognitive impairments following cerebral malaria. Arch Dis Child. 2006;91(2):142–8. doi: 10.1136/adc.2005.077784 16326798 PMC2082712

[4] Molyneux ME, Taylor TE, Wirima JJ, Borgstein A. Clinical features and prognostic indicators in paediatric cerebral malaria: a study of 131 comatose Malawian children. Q J Med. 1989;71(265):441–59. doi: 10.1093/oxfordjournals.qjmed.a068338 2690177

[5] Brewster DR. Neurological sequelae of cerebral malaria in children. Lancet. 1990;336:5. 10.1016/0140-6736(90)92498-7 1977027

[6] Olumese PE, Gbadegesin RA, Adeyemo AA, Brown B, Walker A. Neurological features of cerebral malaria in Nigerian children. Ann Trop Paediatr. 1999;19(4):321–5. doi: 10.1080/02724939992149 10716024

[7] Oguche S, Omokhodion SI, Adeyemo AA, Olumese PE. Low plasma bicarbonate predicts poor outcome of cerebral malaria in Nigerian children. West Afr J Med. 2002;21(4):276–9. doi: 10.4314/wajm.v21i4.27996 12665262

[8] Opoka RO, Bangirana P, Boivin MJ, John CC, Byarugaba J. Seizure activity and neurological sequelae in Ugandan children who have survived an episode of cerebral malaria. Afr Health Sci. 2009;9(2):75–81. 19652740 PMC2707052

[9] Datta D, Gopinadhan A, Soto A, Bangirana P, Opoka RO, Conroy AL, et al. Blood biomarkers of neuronal injury in paediatric cerebral malaria and severe malarial anaemia. Brain Commun. 2023;5(6):fcad323. doi: 10.1093/braincomms/fcad323 38075948 PMC10710298

[10] Bondi FS. The incidence and outcome of neurological abnormalities in childhood cerebral malaria: a long-term follow-up of 62 survivors. Trans R Soc Trop Med Hyg. 1992;86(1):17–9. doi: 10.1016/0035-9203(92)90420-h 1566292

[11] van Hensbroek MB, Palmer A, Jaffar S, Schneider G, Kwiatkowski D. Residual neurologic sequelae after childhood cerebral malaria. J Pediatr. 1997;131(1 Pt 1):125–9. doi: 10.1016/s0022-3476(97)70135-5 9255203

[12] Idro R, Karamagi C, Tumwine J. Immediate outcome and prognostic factors for cerebral malaria among children admitted to Mulago Hospital, Uganda. Ann Trop Paediatr. 2004;24(1):17–24. doi: 10.1179/027249304225013240 15005962

[13] Birbeck GL, Molyneux ME, Kaplan PW, Seydel KB, Chimalizeni YF, Kawaza K, et al. Blantyre Malaria Project Epilepsy Study (BMPES) of neurological outcomes in retinopathy-positive paediatric cerebral malaria survivors: a prospective cohort study. Lancet Neurol. 2010;9(12):1173–81. doi: 10.1016/S1474-4422(10)70270-2 21056005 PMC2988225

[14] Alabi AO, Onigbinde MO, Ojuawo A, Medewase VIJ-, Alabi GO, Oladibu OT, et al. Bedside prognostic indicators of fatal outcome among children with cerebral malaria at a tertiary Nigerian hospital. JCDR. 2023. doi: 10.7860/jcdr/2023/56809.17291

[15] Chastang KM, Imam R, Sherman MG, Olowojesiku R, Mukadam AM, Seydel KB, et al. Temporal trends of blood glucose in children with cerebral malaria. Am J Trop Med Hyg. 2023;108(6):1151–6. doi: 10.4269/ajtmh.23-0022 37068750 PMC10540124

[16] Namazzi R, Batte A, Opoka RO, Bangirana P, Schwaderer AL, Berrens Z, et al. Acute kidney injury, persistent kidney disease, and post-discharge morbidity and mortality in severe malaria in children: a prospective cohort study. EClinicalMedicine. 2022;44:101292. doi: 10.1016/j.eclinm.2022.101292 35198918 PMC8850340

[17] Conroy AL, Opoka RO, Bangirana P, Idro R, Ssenkusu JM, Datta D, et al. Acute kidney injury is associated with impaired cognition and chronic kidney disease in a prospective cohort of children with severe malaria. BMC Med. 2019;17(1):98. doi: 10.1186/s12916-019-1332-7 31109328 PMC6528242

[18] Page MJ, McKenzie JE, Bossuyt PM, Boutron I, Hoffmann TC, Mulrow CD, et al. The PRISMA 2020 statement: an updated guideline for reporting systematic reviews. BMJ. 2021;1(372). 10.1136/bmj.n71 PMC8005924 33782057

[19] Vevea JL, Coburn KM. Maximum-likelihood methods for meta-analysis: a tutorial using R. GPIR. 2015;18(3):329–47. doi: 10.1177/1368430214558311

[20] Gegenfurtner A. Comparing two handbooks of meta-analysis: review of Hunter & Schmidt, Methods of meta-analysis: correcting error and bias in research findings, and Borenstein, Hedges, Higgins, and Rothstein, Introduction to meta-analysis. Springer; 2011.

[21] Ryan R, Cochrane Consumers and Communication Review Group. ‘Cochrane Consumers and Communication Review Group: data synthesis and analysis’; 2013. 10.1111/jebm.12066 24325413

[22] Campbell M, McKenzie JE, Sowden A, Katikireddi SV, Brennan SE, Ellis S, et al. Synthesis without meta-analysis (SWiM) in systematic reviews: reporting guideline. BMJ. 2020;368:l6890. doi: 10.1136/bmj.l6890 31948937 PMC7190266

[23] Borenstein M, Hedges LV, Higgins JPT, Rothstein H. Heterogeneity in meta-analysis. In: The handbook of research synthesis and meta-analysis;2019.

[24] Huedo-Medina TB, Sánchez-Meca J, Marín-Martínez F, Botella J. Assessing heterogeneity in meta-analysis: Q statistic or I² index? Psychol Methods. 2006;11(2):193. 16784338 10.1037/1082-989X.11.2.193

[25] Duval S, Tweedie R. Trim and fill: a simple funnel-plot-based method of testing and adjusting for publication bias in meta-analysis. Biometrics. 2000;56(2):455–63. doi: 10.1111/j.0006-341x.2000.00455.x 10877304

[26] Dettori JR, Norvell DC, Chapman JR. Fixed-effect vs random-effects models for meta-analysis: 3 points to consider. Glob Spine J. 2022;12(7):2. 10.1177/21925682221110527 PMC9393987 35723546

[27] Schmutzhard E, Gerstenbrand F. Cerebral malaria in Tanzania. Its epidemiology, clinical symptoms and neurological long term sequelae in the light of 66 cases. Trans R Soc Trop Med Hyg. 1984;78(3):351–3. doi: 10.1016/0035-9203(84)90118-4 6380023

[28] Ahmad SH, Moonis R, Kidwai T, Khan TA, Khan HM, Shahab T. Cerebral malaria in children. Indian J Pediatr. 1986;53:5. 3531002 10.1007/BF02760427

[29] Schapira A, Solomon T, Julien M, Macome A, Parmar N, Ruas I, et al. Comparison of intramuscular and intravenous quinine for the treatment of severe and complicated malaria in children. Trans R Soc Trop Med Hyg. 1993;87(3):299–302. doi: 10.1016/0035-9203(93)90136-e 8236398

[30] Walker O, Salako LA, Omokhodion SI, Sowunmi A. An open randomized comparative study of intramuscular artemether and intravenous quinine in cerebral malaria in children. Trans R Soc Trop Med Hyg. 1993;87(5):564–6. doi: 10.1016/0035-9203(93)90092-5 8266412

[31] Steele RW, Baffoe-Bonnie B. Cerebral malaria in children. Pediatr Infect Dis J. 1995;14(4):281–5. doi: 10.1097/00006454-199504000-00007 7603809

[32] Newton CR, Marsh K, Peshu N, Kirkham FJ. Perturbations of cerebral hemodynamics in Kenyans with cerebral malaria. Pediatr Neurol. 1996;15(1):41–9. doi: 10.1016/0887-8994(96)00115-4 8858700

[33] Crawley J, Smith S, Kirkham F, Muthinji P, Waruiru C, Marsh K. Seizures and status epilepticus in childhood cerebral malaria. QJM. 1996;89(8):591–7. doi: 10.1093/qjmed/89.8.591 8935480

[34] Allen SJ, O’Donnell A, Alexander ND, Clegg JB. Severe malaria in children in Papua New Guinea. QJM. 1996;89(10):779–88. doi: 10.1093/qjmed/89.10.779 8944234

[35] Genton B, al-Yaman F, Alpers MP, Mokela D. Indicators of fatal outcome in paediatric cerebral malaria: a study of 134 comatose Papua New Guinean children. Int J Epidemiol. 1997;26(3):670–6. doi: 10.1093/ije/26.3.670 9222795

[36] Assimadi JK, Gbadoe AD, Atakouma DY, Agbénowossi K, Lawson-Evi K, Gayibor A. Paludisme sévère de l’enfant au Togo. Arch Pédiatr. 1998. 10.1016/s0929-693x(99)80048-7 9885736

[37] Crawley J, Smith S, Muthinji P, Marsh K, Kirkham F. Electroencephalographic and clinical features of cerebral malaria. Arch Dis Child. 2001;84(3):247–53. doi: 10.1136/adc.84.3.247 11207176 PMC1718702

[38] Idro R, Otieno G, White S, Kahindi A, Fegan G, Ogutu B, et al. Decorticate, decerebrate and opisthotonic posturing and seizures in Kenyan children with cerebral malaria. Malar J. 2005;4:57. doi: 10.1186/1475-2875-4-57 16336645 PMC1326205

[39] Ngoungou EB, Dulac O, Poudiougou B, Druet-Cabanac M, Dicko A, Mamadou Traore A, et al. Epilepsy as a consequence of cerebral malaria in area in which malaria is endemic in Mali, West Africa. Epilepsia. 2006;47(5):873–9. doi: 10.1111/j.1528-1167.2006.00558.x 16686652

[40] Medana IM, Idro R, Newton CRJC. Axonal and astrocyte injury markers in the cerebrospinal fluid of Kenyan children with severe malaria. J Neurol Sci. 2007;258(1–2):93–8. doi: 10.1016/j.jns.2007.03.005 17459417

[41] Ahmed A, Ali SMI. Neurological cases admitted into a General Pediatrics Ward. Pak J Med Sci. 2008;24(1).

[42] Casals-Pascual C, Idro R, Gicheru N, Gwer S, Kitsao B, Gitau E, et al. High levels of erythropoietin are associated with protection against neurological sequelae in African children with cerebral malaria. Proc Natl Acad Sci U S A. 2008;105(7):2634–9. doi: 10.1073/pnas.0709715105 18263734 PMC2268188

[43] Dondorp AM, Fanello CI, Hendriksen ICE, Gomes E, Seni A, Chhaganlal KD, et al. Artesunate versus quinine in the treatment of severe falciparum malaria in African children (AQUAMAT): an open-label, randomised trial. Lancet. 2010;376(9753):1647–57. doi: 10.1016/S0140-6736(10)61924-1 21062666 PMC3033534

[44] Bhanushali M, Taylor TE, Molyneux ME, Sapuwa M, Mwandira E, Birbeck GL. Evoked potentials in pediatric cerebral malaria. Neurol Int. 2011;3(3):e14. doi: 10.4081/ni.2011.e14 22368773 PMC3286154

[45] Manning L, Laman M, Law I, Bona C, Aipit S, Teine D, et al. Features and prognosis of severe malaria caused by Plasmodium falciparum, Plasmodium vivax and mixed Plasmodium species in Papua New Guinean children. PLoS One. 2011;6(12):e29203. doi: 10.1371/journal.pone.0029203 22216212 PMC3245265

[46] Oluwayemi OI, Brown BJ, Oyedeji OA, Adegoke SA, Adebami OJ, Oyedeji GA. Clinical and laboratory predictors of outcome in cerebral malaria in suburban Nigeria. J Infect Dev Ctries. 2013;7(8):600–7. doi: 10.3855/jidc.2769 23949295

[47] Oluwayemi IO, Brown BJ, Oyedeji OA, Oluwayemi MA. Neurological sequelae in survivors of cerebral malaria. Pan Afr Med J. 2013;15:88. doi: 10.11604/pamj.2013.15.88.1897 24198884 PMC3810154

[48] Mergani A, Khamis AH, Fatih Hashim EL, Gumma M, Awadelseed B, Elwali NEMA, et al. Pattern and predictors of neurological morbidities among childhood cerebral malaria survivors in central Sudan. J Vector Borne Dis. 2015;52(3):239–44. doi: 10.4103/0972-9062.166270 26418655

[49] Hawkes MT, Conroy AL, Opoka RO, Hermann L, Thorpe KE, McDonald C, et al. Inhaled nitric oxide as adjunctive therapy for severe malaria: a randomized controlled trial. Malar J. 2015;14:421. doi: 10.1186/s12936-015-0946-2 26510464 PMC4625637

[50] Oninla S, Ogunro P, Oninla O, Kayode O. Childhood cerebral malaria in Nigeria: clinical features, treatment and outcome. IJTDH. 2016;12(4):1–12. doi: 10.9734/ijtdh/2016/22447

[51] O’Brien NF, Mutatshi Taty T, Moore-Clingenpeel M, Bodi Mabiala J, Mbaka Pongo J, Ambitapio Musungufu D, et al. Transcranial Doppler ultrasonography provides insights into neurovascular changes in children with cerebral malaria. J Pediatr. 2018;203:116-124.e3. doi: 10.1016/j.jpeds.2018.07.075 30224088

[52] Conroy AL, Opoka RO, Bangirana P, Namazzi R, Okullo AE, Georgieff MK, et al. Parenteral artemisinins are associated with reduced mortality and neurologic deficits and improved long-term behavioral outcomes in children with severe malaria. BMC Med. 2021;19(1):168. doi: 10.1186/s12916-021-02033-1 34315456 PMC8317420

[53] Prasad R, Patel RS, Mishra SP, Singh A, Abhinay A, Singh TB. Cerebrospinal fluid tumor necrosis factor-alpha (TNF-α) levels in children with cerebral malaria. J Trop Pediatr. 2023;69(6):fmad032. doi: 10.1093/tropej/fmad032 37805828

[54] Clark DJ, Bond C, Andrews A, Muller DJ, Sarkisian A, Opoka RO. Admission clinical and EEG features associated with mortality and long-term neurologic and cognitive outcomes in pediatric cerebral malaria. Neurology. 2023. 10.1212/WNL.0000000000207657 PMC10558167 37541845

[55] Carter JA, Mung’ala-Odera V, Neville BGR, Murira G, Mturi N, Musumba C, et al. Persistent neurocognitive impairments associated with severe falciparum malaria in Kenyan children. J Neurol Neurosurg Psychiatry. 2005;76(4):476–81. doi: 10.1136/jnnp.2004.043893 15774431 PMC1739592

[56] Ngoungou EB, Preux P-M. Cerebral malaria and epilepsy. Epilepsia. 2008;49 Suppl 6:19–24. doi: 10.1111/j.1528-1167.2008.01752.x 18754957

[57] Brim R, Mboma S, Semrud-Clikeman M, Kampondeni S, Magen J, Taylor T, et al. Cognitive outcomes and psychiatric symptoms of retinopathy-positive cerebral malaria: cohort description and baseline results. Am J Trop Med Hyg. 2017;97(1):225–31. doi: 10.4269/ajtmh.17-0020 28719298 PMC5508917

