# Supplementary material for: Prevalence and risk factors of gross neurologic deficits in children after severe malaria: A systematic review and meta-analysis
Source: PLoS One. 2026 Mar 17;21(3):e0333258. doi: 10.1371/journal.pone.0333258 (PMC12994846; doi:10.1371/journal.pone.0333258)
Supplement: S1 Protocol — (PDF) [file pone.0333258.s001.pdf]

Okullo et al. Systematic Reviews (2025) 14:76
https://doi.org/10.1186/s13643-025-02785-4

Systematic Reviews

PROTOCOL Open Access

Prevalence and risk factors of gross neurologic deficits in children after severe malaria: a systematic review protocol

Allen Eva Okullo[1,2,3*], Chandy C. John[2,4], Richard Idro[2,5], Andrea L. Conroy[2,4], Alison Annet Kinengyere[3,6], Kevin Ouma Ojiambo[3,7], Caroline Otike[3,8], Simple Ouma[5], Moses Ocan[3,9], Ekwaro A. Obuku[3,7,10] and Michael Boele van Hensbroek[1]

Abstract

Background Children exposed to severe malaria may recover with gross neurologic deficits (GND). Several risk factors for GND after cerebral malaria (CM), the deadliest form of severe malaria, have been identified in children. However, there is inconsistency between previously reported and more recent findings. Although CM patients are the most likely group to develop GND, it is not clear if other forms of severe malaria (non-CM) may also contribute to malaria-related GND. The objective of this systematic review is to synthesize evidence on the prevalence and risk factors for GND in children after severe malaria.

Methods The systematic review will be conducted according to recommendations of the Preferred Reporting Items for Systematic Reviews and Meta-Analyses for Protocols (PRISMA-P). Relevant research articles will be identified using relevant search terms from the following databases: MEDLINE, Embase, Web of Science, and Global Index Medicus (GIM). The articles will be screened at title and abstract and then at full text for inclusion using a priori eligibility criteria. Data extraction will be carried out using a tool developed and optimized in an Excel spreadsheet. Risk of bias will be assessed using appropriate tools including Risk Of Bias In Non-randomized Studies of Exposures (ROBINS-E) and the Cochrane Risk of Bias 2.0 (ROB2) for randomized control trials (RCTs), and where appropriate, publication bias will be assessed using a funnel plot. A random-effects meta-analysis or synthesis without meta-analysis (SWiM) will be performed as appropriate, and the results will be presented in tables and graphs.

Conclusion Findings from this systematic review will inform policymakers on the planning, design, and implementation of interventions targeting the treatment and rehabilitation of GND following severe malaria in children.

Systematic review registration PROSPERO CRD42022297109.

Keywords Gross neurologic deficit, Cerebral malaria, Severe malaria, Prevalence, Risk factors, Children

*Correspondence:
Allen Eva Okullo
allenokullo.c@gmail.com; aokullo@musph.ac.ug
Full list of author information is available at the end of the article

© The Author(s) 2025. **Open Access** This article is licensed under a Creative Commons Attribution-NonCommercial-NoDerivatives 4.0 International License, which permits any non-commercial use, sharing, distribution and reproduction in any medium or format, as long as you give appropriate credit to the original author(s) and the source, provide a link to the Creative Commons licence, and indicate if you modified the licensed material. You do not have permission under this licence to share adapted material derived from this article or parts of it. The images or other third party material in this article are included in the article's Creative Commons licence, unless indicated otherwise in a credit line to the material. If material is not included in the article's Creative Commons licence and your intended use is not permitted by statutory regulation or exceeds the permitted use, you will need to obtain permission directly from the copyright holder. To view a copy of this licence, visit http://creativecommons.org/licenses/by-nc-nd/4.0/.

Background

In 2021, about 3.4 billion (43%) of the world's population had a neurological disorder with neurodevelopmental and paediatric conditions contributing to 80.3 million (18.2%) DALYs [1]. In the same year, an estimated 247 million cases of malaria were reported from 84 malaria-endemic countries globally, with the WHO African region accounting for 95% of this burden [2]. The severe malaria burden is estimated at less than 1% of the total malaria cases diagnosed [3] with under-5-year-old children in sub-Saharan Africa being most at risk of both severe malaria and death from severe malaria [4].

Severe malaria is a multi-syndromic illness complication of *Plasmodium falciparum* infection that manifests in the form of cerebral malaria, severe malaria anaemia, respiratory distress, malaria with seizures, and malaria with prostration among under-5-year-old children [4]. Survivors of cerebral malaria (CM) may develop neurological sequelae, some of which may cause lifelong disabilities [4–8]. These sequelae may include gross neurologic deficits (GND) such as cortical blindness, ataxia, hemiparesis, deafness, and abnormal movement disorders [4–6] and are often more common among children, with the prevalence ranging from 6 to 29% at hospital discharge [4–6, 9, 10]. Survivors with GND may die within a few months after discharge [5]. Given the burden of GND, its deleterious impact on child survival and health-related quality of life, and an unclear picture of the prevalence and risk factors for GND in children after severe malaria without overt neurological complications on admission, this systematic review will help to inform clinicians and policymakers to control and rehabilitate GND after severe malaria.

Several studies have reported significant declines in the proportion of children with persistent neurological sequelae after CM within the first few months post-discharge [9, 11–13]. Some neurologic sequelae, like cortical blindness, may be transient [14–17], while others may persist, including hemiplegia, paresis, extrapyramidal features, and epilepsia [5, 17]. In a study conducted in The Gambia [9], the proportion of children with neurological sequelae after CM had reduced from 23.3% at discharge to 8.6% 1 month after discharge, with a further decline to 4.4% at 6 months. Similarly, in Uganda, the proportion of children with neurological sequelae after CM declined from 28.2% at discharge to 9.5% at 3 months after discharge, with all children recovering by 6-month post-discharge [12]. Consequences of these sequelae include reduced quality of life and an increase in disability-adjusted life years [17].

Risk factors for neurological sequelae following severe malaria among children which have been reported include deep and prolonged coma, multiple seizures [4–6, 9, 10], acute seizures [8], intracranial hypertension [9, 13, 18–20], male gender, a higher admission temperature [21], age younger than 3 years [18], acute kidney injury (AKI) [22–24], and a biphasic clinical course marked by recovery of consciousness followed by recurrent convulsions and coma [13]. Also, the antimalarial artesunate has previously been associated with ataxia and slurring of speech [25]; however, new evidence, on the contrary, suggests fewer neurologic deficits in children receiving these artemisinin-based treatments [26].

However, recent studies have not been able to confirm the importance of previously associated factors such as hypoglycaemia, anaemia, age, sex, multiplicity of convulsions, and artesunate treatment with persistence of GND after CM [6, 9, 10, 26]. There is a need for evidence on whether this is a true change over time or if it is due to a difference in reporting patterns or analysis. In addition, no systematic review has synthesized evidence on the prevalence or risk factors of GND among children after severe malaria. The prevalence of GND among children with different forms of severe malaria and the most important risk factors will be identified in this synthesis. This will inform the targeting and development of effective treatment protocols to prevent GND among children. The objective of this systematic review is to synthesize the prevalence and risk factors for GND in children after severe malaria.

Methods

This systematic review protocol has been written following the Preferred Reporting Items for Systematic Review and Meta-Analysis Protocols 2015 guidelines (PRISMA-P) [27]. The protocol is registered in the International Prospective Register of Systematic Reviews (PROSPERO), registration number CRD42022297109.

Review question

Primary review question

What is the prevalence of GND among children after severe malaria in malaria-affected regions globally?

Secondary review question

What are the risk factors for GND among children after severe malaria in malaria-affected regions globally?

Hypothesis

Several factors including but not limited to deep/prolonged coma, multiple seizures, hypoglycaemia, clinical features of intracranial hypertension, anaemia, age, sex, malnutrition (macro and micronutrient deficiency), hyperpyrexia, acute kidney injury, chronic kidney disease, hemolysis, endothelial activation, central nervous system inflammation, parasitic biomass, and endothelial

dysregulation may increase the risk of GND among children with severe malaria.

Eligibility criteria

Peer-reviewed articles will be selected according to the following eligibility criteria.

Inclusion criteria

- Articles reporting on the prevalence of GND after WHO-defined severe malaria: GND refers to an abnormal neurologic function of a specific body part arising from injury to the brain, spinal cord, muscles, or nerves that feed the affected area. GND may include motor impairments and movement disorders (ataxia, tremor, dystonia, cranial nerve palsies, mono paresis, hemiparesis or quadriparesis, monoplegia, hemiplegia, and quadriplegia), speech or language impairments, and hearing or visual impairment.
- Articles reporting on GND after severe malaria from the earliest published study in 1946 to date: Severe malaria in this study will be defined as the presence of *P. falciparum* asexual parasitaemia on a blood smear along with one or more of the following: impaired consciousness (a Blantyre coma score of < 3), acidosis or respiratory distress, hypoglycaemia, severe malarial anaemia, multiple convulsions, prostration, renal impairment, jaundice, pulmonary oedema, significant bleeding, shock, or hyperparasitaemia with no other confirmed cause for the symptoms or signs [4].
- Articles reporting on risk factors for GND after severe malaria: Risk factors will include but are not limited to multiple seizures, deep/prolonged coma, hypoglycaemia, clinical features of intracranial/ hypertension, anaemia, age, sex, malnutrition (macronutrient and micronutrient deficiency), hyperpyrexia, acute kidney injury, chronic kidney disease, hemolysis, endothelial activation, central nervous system inflammation, parasitic biomass, endothelial dysregulation, and antimalarials like artesunate.
- Articles reporting on children (persons younger than 18 years)
- Articles with cross-sectional, case–control, cohort, randomized controlled trials, quasi-experimental study, and case series designs
- Articles in all languages: Google Translate will be used for articles not in English. These will qualify at title and abstract screening.

Exclusion criteria

- Articles which are editorials, opinions, consensus papers, conference abstracts, guidelines, recommendations, qualitative research, and literature reviews
- Articles whose full text cannot be retrieved by the librarian and information specialist A. A. K. from external sources such as Web of Science, Embase, and Lib-Hub and in consultation with other librarians.

The review will be guided by the following elements: population, intervention/exposure, comparator, study design, setting, and timing (PICOST) as shown in Table 1.

Table 1 Review questions using the PICOST framework

Population	Children (persons younger than 18 years) who had severe malaria. Severe malaria in this study will be defined as the presence of *P. falciparum* asexual parasitaemia on a blood smear along with one or more of the following: impaired consciousness (a Blantyre coma score of < 3), acidosis or respiratory distress, hypoglycaemia, severe malarial anaemia, multiple convulsions, prostration, renal impairment, jaundice, pulmonary oedema, significant bleeding, shock, or hyperparasitaemia with no other confirmed cause for the symptoms or signs
Exposure	The exposure for this study is severe malaria. Different forms of severe malaria are cerebral malaria, severe malaria anaemia, respiratory distress, malaria with seizures, and malaria with prostration
Comparator	None
Outcome	The study outcome is GND. Different forms of GND include motor impairments and movement disorders (ataxia, tremor, dystonia, cranial nerve palsies, mono paresis, hemiparesis or quadriparesis, and monoplegia, hemiplegia, and quadriplegia), speech or language impairments, and hearing or visual impairment
Study design	The review will include cross-sectional studies, cohort studies, case–control studies, randomized controlled trials, diagnostic studies, quasi-experimental study designs, and case series
Setting	Malaria-affected regions globally
Timing/date of publication	1946 (earliest date for the first journal publication on severe malaria) to date

Patient and public involvement

There will be no patient involvement.

Language

There will be no language restriction to this review.

Information sources

The following electronic databases will be searched: MEDLINE, Embase, Web of Science, and Global Index Medicus (GIM). In addition to this, we will search bibliographies of all included studies. We will search for grey literature from the WHO website, institutional repositories, and leading researchers on neurologic deficits after severe malaria.

Search strategy

This review will use a search strategy developed in collaboration with an experienced librarian and information specialist (A. A. K.) skilled in systematic reviews. A. A. K. will perform the article search using search terms together with their synonyms and MeSH (Medical Subject Headings) developed from our review of articles published in peer-reviewed journals on neurological sequelae after severe malaria from the earliest published study to date. The following search terms will be used to identify eligible studies: 'child', 'children', 'pre-school', 'infant', 'infants', 'malaria', 'cerebral', 'severe malaria', 'impaired consciousness', 'acidosis', 'respiratory distress', 'multiple convulsions', 'prostration', 'repeated seizures', 'multiple seizures', 'hypoglycaemia', 'severe anaemia', 'severe anemia', 'renal impairment', 'acute kidney injury', 'jaundice', 'pulmonary oedema', 'significant bleeding', 'abnormal bleeding', 'shock', 'hyperparasitaemia', 'hyperlactataemia', 'nervous system disease', 'brain disease', 'neurologic', 'sequelae', 'complication', 'impairment', 'deficit', 'prognosis', 'risk factors', 'prevalence', 'frequency', 'risk', 'prediction', 'epidemiological', and 'association'. The terms will be combined using Boolean operators "OR" or "AND" and truncation where applicable.

A pilot search conducted in MEDLINE will be tested for precision to ensure a high proportion of appropriate articles are retrieved. Our initial screening of an electronic search of 755 titles and abstracts performed on 6 September 2023, in MEDLINE, yielded 37 (4.9%) articles as potentially eligible (Table 2).

The search string from MEDLINE has been included as an additional file. The final validated search string from MEDLINE will be adapted to the syntax of other databases, that is, Embase, Web of Science, and Global Index Medicus, and these will also be searched as part of the review. The identified articles will be transferred to EPP-reviewer software.

Selection process and data management

All identified references will be imported into EPPI-Reviewer (eppi.ioe.ac.uk/EPPIReviewer-Web) with full texts and bibliography details. We shall then run a duplicate search and remove all duplicates. A screening tool will be developed based on the study's inclusion and exclusion criteria. This will be tested for its robustness on 5% of the articles, among four reviewers. Titles and abstracts will be screened for potential relevance by four independent reviewers (A. E. O., K. O. O., C. O., and S. O.) in duplicate, that is, A. E. O. and K. O. O., C. O. and S. O., A. E. O. and C. O., K. O. O. and S. O., A. E. O. and S. O., and K. O. O. and C. O., using a priori inclusion and exclusion criteria. Any disagreements between the reviewers will be resolved through consensus after discussion. The selected articles will then be screened in full text independently by the four reviewers working in duplicate. Any disagreements will be resolved by discussion. Screening and coding will be done using EPPI-Reviewer software. The total number of studies included and excluded as well as the reasons for exclusion at each

Table 2 Feasibility of yield of literature of pilot electronic search for prevalence and risk factors of gross neurologic deficits among children after severe malaria in MEDLINE

Search number (database)	Search terms (and date)	Number of hits[a] (relevant)
#1 MEDLINE	exp child/ or (children or pre-school or infant or infants).ti,ab,kf. AND exp Malaria, Cerebral/ or ((cerebral adj3 malaria) or severe malaria or impaired consciousness or acidosis or respiratory distress or multiple convulsions or prostration or repeated seizures or multiple seizures or hypoglycaemia or severe anaemia or severe anemia or renal impairment or acute kidney injury or jaundice or pulmonary oedema or significant bleeding or abnormal bleeding or shock or hyperparasitaemia or hyperlactataemia).ti,ab,kf. AND ("nervous system disease*" or "Brain Disease*" or ("neuro* complicat*" or "neuro* impair*" or "neuro* deficit*" or "neuro* sequelae")).ti,ab,kf. AND exp Prognosis/ or exp Risk Factors/ or prevalence/ or (prevalence or frequen* or risk or prognos* or predict* or epidemiological or associat*).ti,ab,kf	[a]755 ([b]37, 4.9%)

[a] Number of article titles and abstracts as of 6 September 2023

[b] Sorted by relevance and initial screening of titles and abstracts

stage of the selection process will be presented in a PRISMA flow chart. The reasons for exclusion or inclusion will also be presented in the main text. A list of studies that were excluded at full-text screening along with their reasons for exclusion will be provided as an appendix to the final manuscript.

Data abstraction

A data abstraction tool will be developed in EPPI-Reviewer and pretested using 5% of the selected articles. The data abstraction tool will include key variables for abstraction from each article, namely: study characteristics (author, year of publication, year of data collection, title, citation, institution, country, language, source of funding), study design, population characteristics (sample size, age at diagnosis of severe malaria), type of gross neurologic deficit (including author specific definitions), duration of gross neurologic deficit, type of assessment for gross neurologic deficit, risk factors, measures of association (such as risk ratios, odds ratios), and prevalence of gross neurologic deficit. The reviewers (A. E. O., K. O. O., C. O., S. O.) will independently abstract the data, and any disagreements will be resolved through discussion.

Preliminary findings

To determine the feasibility of this review, we have listed five studies from the MEDLINE search that meet the eligibility criteria after initial screening of titles and abstracts (Table 3).

These studies were published between 1990 and 2013 and were conducted among children who had previously been admitted to hospitals in Kenya [6], The Gambia [9, 13], and Nigeria [10, 15]. These studies reported prevalence and risk factors for neurologic sequelae after childhood cerebral malaria. GND within these studies ranged from 12 to 23.8% which is the outcome of the study. Risk factors for GND from these studies included prolonged coma, depth of coma, multiple convulsions, hypoglycaemia, previous seizures, multiple seizures, and focal neurological signs observed during admission.

Table 3 Preliminary findings of potentially eligible studies

Author	Year	Country	Design	Population
Idro et al. [5]	2006	Kenya	Cross-sectional	Children
Van Hensbroek et al. [8]	1997	The Gambia	Prospective cohort	Children
Oluwayemi et al. [9]	2013	Nigeria	Cross-sectional	Children
Brewster et al. [12]	1990	The Gambia	Prospective cohort	Children
Bondi [14]	1992	Nigeria	Prospective cohort	Children

Risk-of-bias assessment

Four members of the team (A. E. O., K. O. O., C. O., and S. O.) shall independently assess the methodological quality of the included studies. Risk-of-bias assessment shall be carried out using ROBINS-E ('Risk Of Bias In Non-randomized Studies of Exposures') tool for non-randomized controlled trials (https://www.riskofbias.info/welcome/robins-e-tool). Using the ROBINS-E, bias will be assessed as a judgment (low risk of bias, some concerns, high risk of bias, very high risk of bias) using seven domains. These domains are risk of bias due to confounding, risk of bias arising from the measurement of the exposure, risk of bias in the selection of participants into the study (or into the analysis), risk of bias due to postexposure interventions, risk of bias due to missing data, risk of bias arising from the measurement of the outcome, and risk of bias in the selection of the reported result.

The Cochrane Risk of Bias 2.0 (ROB2) tool will be used for randomized control trials (RCTs) (https://methods.cochrane.org/bias/resources/cochrane-risk-bias-tool). Using this tool, bias will be assessed as a judgment (high, low, or unclear) for individual elements using five domains, namely: selection bias, reporting bias, performance bias, attrition bias, and detection, and other biases [28]. Other study designs will be assessed using the Qualitative Assessment Tool for Quantitative Studies (Effective Public Health Practice Project, https://merst.ca/ephpp/). This tool provides an overall rating (weak, moderate, or strong) based on an appraisal of eight domains: selection bias, study design, confounders, blinding, data collection methods, withdrawals and dropouts, intervention integrity, and analysis.

Publication bias

We will assess the risk of publication bias in the included articles by using the asymmetry of funnel plots. This is a rank-based data augmentation technique that has proved to be accurate for assessing publication bias due to missing data or studies [29]. In the absence of missing studies, the shape of the scatter plot should resemble a symmetrical inverted funnel with a wide base (consisting of small studies with large effect estimate variability) and a narrow top (consisting of large studies with small effect estimate variability) [30]. The presence of large 'holes' — often seen close to the bottom — or asymmetry in the plot indicates publication bias, though these holes may have other causes, such as study heterogeneity [31].

Assessment of strength and confidence of cumulative evidence

The strength of evidence will be graded based on the assessment of study limitations, directness, consistency, precision, and reporting bias. The overall quality of evidence will be assessed using a modified Grading, Recommendation, Assessment Development, and Evaluation (GRADE) framework. In the GRADE method, the quality of evidence is rated for each key outcome as 'high', 'moderate', 'low', or 'very low'. Observational studies start at low quality and may be upgraded, while randomized trials are set at high quality and may be downgraded. We will develop a summary of findings tables and assess the confidence in the effect estimates and strength of associations based on the determined quality of evidence.

Heterogeneity

The I^2 statistic will be used to assess the level of statistical heterogeneity in the articles. The I^2 statistic will show the percentage (%) of heterogeneity attributable to between-study variation [32]. Heterogeneity will be categorized as low ($I^2=25\%$) (low), moderate ($I^2=50$), and high ($I^2>75\%$) [28].

Data synthesis

The prevalence of GND will be synthesized using the Freeman-Tukey double arcsine transformation approach. Sub-group analyses will be performed to compare the prevalence of GND by type of severe malaria (CM vs non-CM), age group, and year of publication.

If the data on specific risk factor is adequate, we shall estimate the effect size (risk ratios or odds ratios) for each separately. Effect sizes will be pooled statistically using inverse variance-weighted random effects meta-analysis, using the metan command in Stata v18 [33]. However, if the data is inadequate, we shall perform a synthesis without meta-analysis (SWiM) [34].

The synthesis will further be in the form of a summary of findings tables and forest plots where applicable using the Stata version 18. This will follow the format of the Cochrane Consumers and Communication Review Group [35]. Outcome data will be presented as frequencies and percentages. Prevalence values and measures of association such as risk ratios and odds ratios will be presented as appropriate. We will organize and tabulate results to identify associations which will be described narratively. We will describe the included articles and group articles according to study design, exposure, and outcome. We shall use both narrative and quantitative synthesis.

Sensitivity analysis

We will examine the sensitivity of findings by the risk-of-bias status (low risk versus some concerns or high risk).

Discussion

Neurological disorders (deficits) are the leading cause of DALYs and the second leading cause of death, estimated at 9 million deaths per year, globally [36]. In 2016, 52.9 million children younger than 5 years had developmental disabilities with 95% coming from low- and middle-income countries [36]. These disorders can present in the form of cognitive impairments, behavioural impairments, communication impairments, or physical functioning limitations (gross neurological deficits) [37]. They may result from some diseases such as cerebrovascular disease, maternal causes, conditions arising in the perinatal period, nutritional deficiencies, and injuries as well as from infections such as poliomyelitis, meningitis, and malaria [37].

Lack of prompt and effective management of severe malaria can lead to a consequence of lifelong neurological disabilities in some survivors [2] termed neurological sequelae [9, 13, 17]. Cerebral malaria is the most severe neurological complication of infection with *P. falciparum* malaria [38] and accounts for 10% of all severe malaria episodes among under-5 years old [39] and is associated with almost all neurocognitive sequelae [40].

The global annual incidence of severe malaria has been estimated at approximately two million cases [4] with under-5-year-old children in sub-Saharan Africa being most at risk of severe malaria [2]. In areas of high endemicity, severe malaria mainly occurs among children under 5 years, while in areas of lower endemicity, it occurs in both children and adults [4]. In areas with intense transmission (high inoculation rates), severe anaemia is more predominant, while in places with low transmission, cerebral malaria in slightly older children is predominant [4, 41, 42].

Persons living with neurological deficits face several challenges such as stigma, discrimination, and human rights violations [36, 43]. Caregivers and children with neurologic deficits after severe malaria face social isolation which hinders them from engaging in community activities. Caregivers have reported being mocked and shamed by the community who believe their children are bewitched [43]. Furthermore, children with these deficits face disability stigma in classrooms, especially in rural communities where there is a lack of individualized care, leading to school dropouts [43]. Caretakers of children with neurological disability are further hampered by financial challenges due to the costs of raising physically handicapped children which further limits access to treatment rehabilitation services [44]. Challenges to

the care of children occur when a child has functional limitations and long-term dependence due to disability which affects not only the child but also the entire family [44]. This poses significant psychosocial and socio-economic challenges to parents especially parents who do not have good knowledge about the condition and who do not know how to address problems of the affected children [44].

The significant burden of neurological deficits or disorders especially in LMICs presents a crucial need for research on both prevalence and risk factors among the diseases and infections associated with these deficits. This will inform both clinicians and policymakers to help in the development of treatment protocols aimed at the prevention and rehabilitation of neurologic deficits.

Conclusion

This systematic review will synthesize evidence on the prevalence and risk factors for GND after severe malaria among children. This will provide information on the burden of GND among children after severe malaria which will guide policymakers in targeting interventions for children at the highest risk of GND after severe malaria. This systematic review will also provide clarity on the most important risk factors for GND after severe malaria in children which will inform the selection, design, deployment, and establishment of interventions targeting early detection, prevention, and rehabilitation of GND following severe malaria among children. This systematic review will thus provide comprehensive evidence on the magnitude and risk factors of GND after severe malaria to inform policymakers and clinicians in the control and rehabilitation efforts for GND associated with severe malaria among children.

Anticipated methodological limitations

There may be a limitation in identifying all relevant studies on the prevalence and risk factors for GND among children after severe malaria due to grey literature beyond the reach of the review team. This will be addressed by the use of publication bias which will aid interpretation of the study findings.

Abbreviations

DALYS	Disability-adjusted life years
GND	Gross neurologic deficit
GRADE	Grading, Recommendation, Assessment, Development and Evaluation
LMICs	Low- and middle-income countries
PICOST	Population, intervention/exposure, comparator, outcome, study design, and timing
PRISMA-P	Preferred Reporting Items for Systematic Reviews and Meta-Analysis Protocols
PROSPERO	International Prospective Register of Systematic Reviews
RCT	Randomized controlled trials
ROBINS-E	Risk of Bias in Non-randomized Studies of Exposures

Supplementary Information

The online version contains supplementary material available at https://doi.org/10.1186/s13643-025-02785-4.

Additional file 1. PRISMA-P 2015 Checklist.

Additional file 2. Search string in MEDLINE.

Acknowledgements

I acknowledge the University of Amsterdam Medical Centers, University of Amsterdam, and the Africa Center for Systematic Reviews and Knowledge Translation for all the support and guidance in writing this protocol.

Authors' contributions

AEO drafted the initial protocol. CCJ, RI, AC, KOO, CO, SO, AK, MO, EAO, and MBVH edited the draft protocol. All authors have reviewed the final systematic review protocol. All authors read and approved the final manuscript.

Funding

This systematic review protocol has been conducted with support from grants from the Fogarty International Center (D43 TW010928) and the National Institute of Neurological Disorders and Stroke (R01 NS055349).

Data availability

This section is not applicable since this is a systematic review protocol with no results yet.

Declarations

Ethics approval and consent to participate

Ethics approval for this systematic review protocol has not been sought as this review will involve a synthesis of published and unpublished studies that were ethically approved. Hence, it does not require ethical approval.

Consent for publication

Not applicable.

Competing interests

The authors declare that they have no competing interests.

Author details

[1]Department of Paediatrics, Amsterdam University Medical Centers, University of Amsterdam, P. O. Box 22660, Amsterdam 1100 DD, The Netherlands. [2]Global Health Uganda, Plot 667 Mawanda Road, P. O. Box 33842, Kampala, Uganda. [3]Africa Center for Systematic Reviews & Knowledge Translation, College of Health Sciences, Makerere University, P. O. Box 7072, Kampala, Uganda. [4]Ryan White Center for Pediatric Infectious Diseases and Global Health, Indiana University School of Medicine, 1044 W. Walnut Street, Rm 402D, Indianapolis, IN 46202, USA. [5]Department of Pediatrics and Child Health, School of Medicine, College of Health Sciences, Makerere University, P. O. Box 7072, Kampala, Uganda. [6]Sir Albert Cook Library, College of Health Sciences, Makerere University, P. O. Box 7072, Kampala, Uganda. [7]Clinical Epidemiology Unit, School of Medicine, College of Health Sciences, Makerere University, P.O Box 7072 Kampala, Uganda. [8]Joint Clinical Research Centre, P. O. Box 10005, Kampala, Uganda. [9]Department of Pharmacology & Therapeutics, School of Biomedical Sciences, College of Health Sciences, Makerere University, P. O. Box 7072, Kampala, Uganda. [10]Faculty of Epidemiology & Population Health, London School of Hygiene & Tropical Medicine, WCETH11, Keppel Street, London, UK.

Received: 20 September 2023 Accepted: 7 February 2025
Published online: 03 April 2025

References

1. GBD 2021 Nervous System Disorders Collaborators. Global, regional, and national burden of disorders affecting the nervous system, 1990–2021: a systematic analysis for the Global Burden of Disease Study 2021. Lancet Neurol. 2024;23:37.

2. WHO. World Malaria Report. 2022.
3. Garrido-Cardenas JA, González-Cerón L, García-Maroto F, Cebrián-Carmona J, Manzano-Agugliaro F, Mesa-Valle CM. Analysis of fifty years of severe malaria worldwide research. Pathogens. 2023;12(3):373.
4. WHO. Severe malaria. Trop Med Int Health. 2014;19:124.
5. Trivedi S, Chakravarty A. Neurological complications of malaria. Curr Neurol Neurosci Rep. 2022;22:15.
6. Idro R, Carter JA, Fegan G, Neville BGR, Newton CRJC. Risk factors for persisting neurological and cognitive impairments following cerebral malaria. Arch Dis Child. 2006;91:7.
7. Idro R, Kakooza-Mwesige A, Balyejjussa S, et al. Severe neurological sequelae and behaviour problems after cerebral malaria in Ugandan children. BMC Res Notes. 2010;3:104.
8. Potchen MJ, Birbeck GL, DeMarco JK, Kampondeni SD, Beare N, Molyneux ME, Taylor TE. Neuroimaging findings in children with retinopathy-confirmed cerebral malaria. Eur J Radiol. 2010;74(1):7.
9. van Hensbroek MB, Palmer A, Jaffar S, Schneider G, Kwiatkowski D. Residual neurologic sequelae after childhood cerebral malaria. J Pediatr. 1997;131:5.
10. Oluwayemi IO, Brown BJ, Oyedeji OA, Oluwayemi MA. Neurological sequelae in survivors of cerebral malaria. Pan Afr Med J. 2013;15:88.
11. Opoka RO, Bangirana P, Boivin MJ, John CC, Byarugaba J. 75–81, Seizure activity and neurological sequelae in Ugandan children who have survived an episode of cerebral malaria. Afr Health Sci. 2009;9:7.
12. Boivin MJ, Bangirana P, Byarugaba J, et al. Cognitive impairment after cerebral malaria in children: a prospective study. Pediatrics. 2007;119:7.
13. Brewster DR. Neurological sequelae of cerebral malaria in children. Lancet. 1990;336:5.
14. Schmutzhard E, Gerstenbrand F. Cerebral malaria in Tanzania. Its epidemiology, clinical symptoms and neurological long term sequelae in the light of 66 cases. Transact R Soc Trop Med Hygiene. 1984;78:3.
15. Bondi FS. The incidence and outcome of neurological abnormalities in childhood cerebral malaria: a long-term follow-up of 62 survivors. Royal Society of Tropical Medicine and Hygiene. 1992;86:3.
16. John CC, Bangirana P, Byarugaba J, Opoka RO, Idro R, Jurek AM, Wu B, Boivin MJ. Cerebral malaria in children is associated with long-term cognitive impairment. Pediatrics. 2008;122:8.
17. Nicoline SN, Villabona-Rueda A, Cottier EK, Huether K, Chipeta J, Stins FM. Pathophysiology and neurologic sequelae of cerebral malaria. Malaria J. 2020;19:266.
18. Molyneux ME, Taylor TE, Wirima JJ, Borgstein A. Clinical features and prognostic indicators in paediatric cerebral malaria: a study of 131 comatose Malawian children. Quarterly Journal of Medicine. 1989;71:19.
19. Crawley J, Smith S, Kirkham F, Muthinji P, Waruiru C, Marsh K. Seizures and status epilepticus in childhood cerebral malaria. Quarterly Journal of Medicine. 1996;89:7.
20. Idro R, Karamagi C, Tumwine J. Immediate outcome and prognostic factors for cerebral malaria among children admitted to Mulago Hospital. Uganda Annals of Tropical Paediatrics. 2004;24:8.
21. Birbeck LG, Beare N, Lewallen S, et al. Identification of malaria retinopathy improves the specificity of the clinical diagnosis of cerebral malaria: findings from a prospective cohort study. Am J Trop Med Hyg. 2010;82:4.
22. Conroy AL, Opoka RO, Bangirana P, Idro R, Ssenkusu JM, Datta D, Hodges JS, Morgan C, John CC. Acute kidney injury is associated with impaired cognition and chronic kidney disease in a prospective cohort of children with severe malaria. BMC Medicine. 2019;17:98.
23. Conroy AL, Datta D, Hoffmann A, Wassmer SC. The kidney-brain pathogenic axis in severe falciparum malaria. Trends Parasitol. 2023;39(3):191–9.
24. Hickson MR, Conroy AL, Bangirana P, Opoka RO, Idro R, Ssenkusu JM, John CC. Acute kidney injury in Ugandan children with severe malaria is associated with long-term behavioral problems. PLoS ONE. 2019;14(12):e0226405.
25. Muller LG, Panosian CB. Ataxia and slurred speech after artesunate treatment for falciparum malaria. N Engl J Med. 1997;336:1328.
26. Conroy AL, Opoka RO, Bangirana P, Namazzi R, Okullo AE, Georgieff MK, Cusick S, Idro R, Ssenkusu JM, John CC. Parenteral artemisinins are associated with reduced mortality and neurologic deficits and improved long-term behavioral outcomes in children with severe malaria. BMC Med. 2021;19(1):168.
27. Moher DSL, Clarke M, Ghersi D, Liberati A, Petticrew M, et al. Preferred Reporting Items for Systematic Review and Meta-Analysis Protocols (PRISMA-P) 2015 statement. Syst Rev. 2015;4(1):1.
28. Higgins JPT, Thomas J, Chandler J et al. Cochrane Handbook for Systematic Reviews of Interventions. 2019: John Wiley & Sons.
29. Ocan M, Loyce N, Ojiambo KO, Kinengyere AA, Apunyo R, Obuku EA. Efficacy of antimalarial herbal medicines used by communities in malaria affected regions globally: a protocol for systematic review and evidence and gap map. BMJ Open. 2023;13(7):e069771.
30. Duval S, Tweedie R. Trim and fill: a simple funnel-plot-based method of testing and adjusting for publication bias in meta-analysis. Biometrics. 2000;56:9.
31. Page MJ, Higgins JP, Sterne JA. Assessing risk of bias due to missing results in a synthesis. Cochrane Handbook for Systematic Reviews of Interventions. 2019;13:34–374.
32. Higgins JP. Commentary: Heterogeneity in meta-analysis should be expected and appropriately quantified. Int J Epidemiol. 2009;37(5):1158–60.
33. Harris RJ, Deeks JJ, Altman DG, Bradburn MJ, Harbord RM, Sterne JAC. metan: fixed- and random-effects meta-analysis. The Stata Journal. 2008;8(1):328.
34. Campbell M. McKenzie, JE, Sowden A, Katikireddi SV, Brenann SE, Ellis S, Hartmann-Boyce J, Ryan R, Shepperd S, Thomas J, Welch V, Thomson H, Synthesis without meta-analysis (SWiM) in systematic reviews: reporting guideline. BMJ Open. 2020;368:l6890.
35. Ryan R. Cochrane Consumers and Communication Review Group: data synthesis and analysis. Cochrane Consumers and Communication Review Group. 2013;2019:216–20.
36. WHO. Intersectoral global action plan on epilepsy and other neurological disorders. 2023.
37. WHO. Neurological disorders public health challenges. 2006.
38. Idro R, Marsh K, John CC, Newton CRJ. Cerebral malaria: mechanisms of brain injury and strategies for improved neurocognitive outcome. Pediatr Res. 2010;68:8.
39. Murphy S, Breman JG. Gaps in the childhood malaria burden in Africa: cerebral malaria, neurological sequelae, anemia, respiratory distress, hypoglycemia and complications of pregnancy. Am J Trop Med Hyg. 2001;64:11.
40. Mohanty S, Taylor TE, Kampondeni S, Potchen MJ, Panda P, Majhi M, Mishra SK, Wassmer SC. Magnetic resonance imaging during life: the key to unlock cerebral malaria pathogenesis? Malaria J. 2014;13:276.
41. Carneiro L, Roca-Feltrer A, Griffin JT, Smith L, Tanner M, Schellenberg JA, Greenwood B, Schellenberg D. Age-patterns of malaria vary with severity, transmission intensity and seasonality in sub-Saharan Africa: a systematic review and pooled analysis. PLoSOne. 2010;5(2):e8988.
42. Roca-Feltrer A, Carneiro L, Schellenberg JRMA. Estimates of the burden of malaria morbidity in Africa in children under the age of 5 years. Tropical Med Int Health. 2008;13(6):12.
43. Boubour A, Mboma S, Võ T, Birbeck LG, Seydel BK, Mallewa M, Chinguo D, Gladstone M, Mohamed S, Thakur TK. "We can't handle things we don't know about": perceived neurorehabilitation challenges for Malawian paediatric cerebral malaria survivors. BMC Pediatrics. 2020;20(1):503.
44. Lawal H, Anyebe EE, Obiako OR, Garba SN. Socio-economic challenges of parents of children with neurological disorders: a hospital-based study in North West Nigeria. Int J Nursing Midwifery. 2014;6(4):9.

Publisher's Note

Springer Nature remains neutral with regard to jurisdictional claims in published maps and institutional affiliations.
