# Supplementary material for: Prevalence and risk factors of gross neurologic deficits in children after severe malaria: A systematic review and meta-analysis
Source: PLoS One. 2026 Mar 17;21(3):e0333258. doi: 10.1371/journal.pone.0333258 (PMC12994846; doi:10.1371/journal.pone.0333258)
Supplement: S1 Table — (DOCX) [file pone.0333258.s003.docx]

**List of excluded studies on full text**

| **No.** | **Author** | **Date** | **DOI** | **Reason** |
| --- | --- | --- | --- | --- |
| 1 | A-Elgadir T. M. E | 2006 | 10.1111/j.1365-3083.2006.01732.x | Outcome |
| 2 | Adegoke S. | 2012 | 10.7196/samj.5240 | Outcome |
| 3 | Adonis-Koffy L. | 2004 | 10.1016/j.arcped.2003.09.037 | Study design |
| 4 | Ahmad S. | 2017 | 10.4084/MJHID.2017.006 | Population |
| 5 | Al-Taiar A. | 2006 | 10.1136/bmj.38959.368819.BE | Outcome |
| 6 | Andoh N. E. | 2021 | 10.3389/fcimb.2021.741370 | Outcome |
| 7 | Andrews A. | 2022 | 10.1016/j.pediatrneurol.2021.10.012 | Outcome |
| 8 | Asma U. | 2014 | 10.3347/kjp.2014.52.6.621 | Population |
| 9 | Bangirana P. | 2016 | 10.1016/j.lindif.2015.01.010 | Outcome |
| 10 | Boivin M. J. | 2011 | 10.1111/j.1365-3156.2010.02704.x | Outcome |
| 11 | Boivin M. J. | 2019 | 10.1097/inf.0000000000002367 | Outcome |
| 12 | Boubour A. | 2019 | 10.1016/j.jns.2019.10.1689 | Duplicate |
| 13 | Carter J. A. | 2005 | 10.1136/jnnp.2004.043893 | Outcome |
| 14 | Chiabi A. | 2004 |  | Outcome |
| 15 | Commey J. | 1994 |  | Outcome |
| 16 | Clarke | 2023 |  | Duplicate cohort |
| 17 | Conroy A. L. | 2021 | 10.1186/s12916-021-02033-1 | Outcome |
| 18 | Conroy A. | 2021 | 10.1016/j.ekir.2021.03.184 | Outcome |
| 19 | Cserti-Gazdewich C. M. | 2012 | 10.1111/bjh.12014 | Outcome |
| 20 | Cusick S. E. | 2016 | 10.3945/ajcn.115.117820 | Outcome |
| 21 | Datta D. | 2016 | 10.4269/ajtmh.abstract2016 | Outcome |
| 22 | Datta D. | 2017 | [10.1093/cid/ciz325](https://doi.org/10.1093/cid/ciz325) | Outcome |
| 23 | Datta D. | 2020 | 10.1093/cid/ciz325 | Duplicate |
| 24 | Datta D. | 2021 | [10.1001/jamanetworkopen.2021.38515](https://dx.doi.org/10.1001/jamanetworkopen.2021.38515) | Outcome |
| 25 | Datta D. | 2023 |  | Duplicate cohort |
| 26 | Day N. | 2007 |  | Outcome |
| 27 | Doumbo O. K. | 2009 | 10.4269/ajtmh.2009.09-0406 | Outcome |
| 28 | Dzeing-Ella A. | 2005 | 10.1186/1475-2875-4-1 | Outcome |
| 29 | Fernander E. M. | 2022 | 10.20411/pai.v7i1.499 | Outcome |
| 30 | Gordeuk V. R. | 2010 |  | Outcome |
| 31 | Hawkes M. | 2011 | [10.1186/1745-6215-12-176](https://dx.doi.org/10.1186/1745-6215-12-176) | Outcome |
| 32 | Hay F. | 2000 | 10.1016/S0929-693X%2800%2900125-1 | Outcome |
| 33 | Hora R. | 2016 | 10.1007/s11011-015-9787-5 | Study design |
| 34 | Ibrahim M. L. | 2007 | 10.1016/j.micinf.2007.02.003 | Outcome |
| 35 | Idro R. | 2007 | [10.1001/jama.297.20.2232](https://dx.doi.org/10.1001/jama.297.20.2232) | Outcome |
| 36 | Idro R. | 2008 | 10.1186/1471-2431-8-5 | Outcome |
| 37 | Llunga-llunga F. | 2016 | 10.1016/j.arcped.2016.01.017 | Outcome |
| 38 | Imbert P | 2002 | 10.1016/s0035-9203(02)90099-4 | Outcome |
| 39 | Issifou S. | 2007 | 10.4269/ajtmh.2007.77.1015 | Outcome |
| 40 | Jain V. | 2013 | [10.1179/204777213X13777615588180](https://dx.doi.org/10.1179/204777213X13777615588180) | Outcome |
| 41 | John C. C. | 2008 | 10.1542/peds.2007-3709 | Outcome |
| 42 | Kampondeni S. D. | 2013 | 10.4269/ajtmh.12-0538 | Outcome |
| 43 | Kariuki S. M. | 2014 | 10.1186/1475-2875-13-365 | Outcome |
| 44 | Kayano A. C. A. V. | 2016 | 10.1128/IAI.01404-15 | Outcome |
| 45 | Kingston H. W. F | 2020 | 10.1093/infdis/jiz568 | Outcome |
| 46 | Laman M. | 2013 | 10.4269/ajtmh.13-0281 | Outcome |
| 47 | Lang B. | 2005 | 10.1086/426512 | Outcome |
| 48 | Lyke K. E. | 2003 | 10.4269/ajtmh.2003.69.253 | Outcome |
| 49 | Maitland K. | 2005 | 10.1016/j.pt.2004.10.010 | Outcome |
| 50 | Martinez-Salazar E. L. | 2014 | 10.1590/0037-8682-0078-2014 | Outcome |
| 51 | Mayor A. | 2011 | 10.1371/journal.pone.0019422 | Outcome |
| 52 | Meremikwu M. M. | 1997 |  | Outcome |
| 53 | Mibei E. K. | 2008 | 10.1111/j.1365-3024.2008.01030.x | Outcome |
| 54 | Molyneux M. E. | 1991 |  | Outcome |
| 55 | Muchohi S. N. | 2001 | 10.1016/s0378-4347(01)00284-5 | Outcome |
| 56 | Nallandhighal S. | 2018 | 10.1093/ofid/ofy209.079 | Outcome |
| 57 | Namazzi R. | 2022 | 10.1093/cid/ciac229 | Outcome |
| 58 | Newton C. R. | 1997 | 10.1016/s0035-9203(97)90207-8 | Outcome |
| 59 | Ngoungou | 2007 |  | Duplicate cohort |
| 60 | Njuguna P. | 2019 | 10.1186/s12916-019-1359-9 | Outcome |
| 61 | Okoromah C. A. | 2011 | 10.1002/14651858.CD004615.pub3 | Systematic Review |
| 62 | Ouma B. J. | 2019 | 10.1097/CCM.0000000000004469 | Outcome |
| 63 | Postels D. G. | 2013 | [10.1016/B978-0-444-53490-3.00006-6](https://dx.doi.org/10.1016/B978-0-444-53490-3.00006-6) | Study Design |
| 64 | Postels D. G. | 2013 | [10.1002/ana.24069](https://dx.doi.org/10.1002/ana.24069) | Outcome |
| 65 | Postels D. G. | 2015 | 10.1002/ana.24477 | Outcome |
| 66 | Rivera-Correa J. | 2019 | 10.1038/s41598-019-51426-z | Outcome |
| 67 | Roca-Feltrer A. | 2008 | 10.1111/j.1365-3156.2008.02076.x | Systematic Review |
| 68 | Sangowawa A. O. | 2014 | 10.1155/2014/974693 | Outcome |
| 69 | Satpathy S. K. | 2004 | 10.1007/bf02723094 | Outcome |
| 70 | Shabani E. | 2015 | 10.1093/cid/ciu735 | Outcome |
| 71 | Sicuri E. | 2011 | 10.1186/1475-2875-12-307 | Outcome |
| 72 | Stepniewska K. | 2001 | 10.1016/s0035-9203(01)90104-x | Systematic Review |
| 73 | Tanwar G. S. | 2011 | 10.1179/1465328111Y.0000000040 | Outcome |
| 74 | Taylor | 1988 |  | Duplicate cohort |
| 75 | Tchinda V. H. M | 2007 | 10.1016/j.actatropica.2007.02.011 | Outcome |
| 76 | Varandas L. | 2000 | 10.1080/1465328002003852 | Outcome |
| 77 | Varandas L. | 2001 | 10.1080/02724930120077781 | Outcome |
| 78 | Vasse C. | 2022 | 10.1016/j.arcped.2022.01.014 | Outcome |
| 79 | Villaverde C. | 2015 |  | Outcome |
| 80 | Waller D. | 1995 | 10.1093/clinids/21.3.577 | Outcome |
| 81 | White N. J. | 1987 | 10.1016/s0140-6736(87)90354-0 | Outcome |
| 82 | Zhao S. Z. | 2011 | 10.1179/146532811X12925735813724 | Systematic Review |
| 83 | Adil M. | 2007 |  | Outcome |
| 84 | Ahmed S. | 2011 |  | Outcome |
| 85 | Akech S. | 2010 | [10.1097/CCM.0b013e3181e81165](https://dx.doi.org/10.1097/CCM.0b013e3181e81165) | Outcome |
| 86 | Assimadi J. K. | 1998 | 10.1016/S0929-693X%2899%2980048-7 | Duplicate |
| 87 | Bangirana P. | 2013 |  | Duplicate |
| 88 | Bangirana P. | 2014 | 10.1093/cid/ciu293 | Outcome |
| 89 | Bangirana P. | 2018 | 10.1371/journal.pone.0191550 | Outcome |
| 90 | Beare N. A. | 2004 | 10.1001/archopht.122.8.1141 | Outcome |
| 91 | Beare N. A. | 2006 | 10.1136/jnnp.2005.083956 | Outcome |
| 92 | Biemba G. | 2000 | 10.1046/j.1365-3156.2000.00506.x | Outcome |
| 93 | Boivin M. J. | 2014 | 10.1097/INF.0000000000000296 | Outcome |
| 94 | Boivin M. J. | 2014 | 10.3109/02699052.2014.892379 | Duplicate |
| 95 | Borgstein A. | 2022 | 10.1186/s12936-022-04080-2 | Outcome |
| 96 | Carter J. A. | 2004 | 10.1111/j.0013-9580.2004.65103.x | Outcome |
| 97 | Carter J. A. | 2005 | 10.1136/jnnp.2004.043893 | Duplicate |
| 98 | Carter J. A. | 2006 | 10.1017/S0012162206000107 | Outcome |
| 99 | Conroy A. L. | 2010 |  | Outcome |
| 100 | Conroy A. L. | 2019 | 10.1186/s12916-019-1291-z | Study design |
| 101 | Conroy A. L. | 2023 | 10.1016/j.pt.2023.01.005 | Study design |
| 102 | Cusick S. E. | 2020 | 10.1093/ajcn/nqaa004 | Outcome |
| 103 | Datta D. | 2021 | 10.1001/jamanetworkopen.2021.38515 | Outcome |
| 104 | Garg R. K. | 1999 |  | Study design |
| 105 | Gomes M. P. | 2009 | 10.1002/bdra.20605 | Study design |
| 106 | Gupta D. | 2001 | 10.1007/BF02722357 | Study design |
| 107 | Gwer S. | 2013 | 10.1016/j.jcrc.2013.09.001 | Population |
| 108 | Hay F. | 2000 | [10.1016/S0929-693X%2800%2900125-1](https://dx.doi.org/10.1016/S0929-693X%2800%2900125-1) | Outcome |
| 109 | Hickson M. R. | 2019 | 10.1371/journal.pone.0226405 | Outcome |
| 110 | Holmberg D. | 2017 | 10.1186/s12936-017-1954-1 | Outcome |
| 111 | Idro R. | 2016 | 10.1186/s12936-016-1233-6 | Outcome |
| 112 | Inocent G. | 2009 | 10.1097/INF.0b013e3181ab489d | Outcome |
| 113 | Jain V. | 2020 | 10.1016/j.actatropica.2020.105675 | Population |
| 114 | Jallow M. | 2012 | 10.1371/journal.pone.0045645 | Outcome |
| 115 | John C. C. | 2003 |  | Study design |
| 116 | John C. C. | 2008 |  | Outcome |
| 117 | Kampondeni S. | 2020 | 10.1097/INF.0000000000002573 | Outcome |
| 118 | Karikari A. | 2021 | 10.1186/s12936-021-03918-5 | Outcome |
| 119 | Khan W. | 2014 | 10.2478/s11686-014-0227-1 | Outcome |
| 120 | Lalloo D. G. | 1996 | 10.4269/ajtmh.1996.55.119 | Population |
| 121 | Langfitt J. T. | 2018 |  | Duplicate |
| 122 | Langfitt J. T. | 2019 | 10.1542/peds.2018-1026 | Outcome |
| 123 | Lopansri B. K. | 2006 |  | Outcome |
| 124 | Mburu W. | 2021 | 10.1093/tropej/fmab091 | Outcome |
| 125 | McDonald C. R. | 2017 | 10.1097/inf.0000000000001382 | Outcome |
| 126 | McGuire W. | 1994 | 10.1038/371508a0 | Outcome |
| 127 | McGuire W. | 1999 | 10.1086/314533 | Outcome |
| 128 | McIntosh H. M. | 2000 | 10.1002/14651858.CD000527 | Study design |
| 129 | Medana I. M. | 2002 | 10.1086/339009 | Population |
| 130 | Medana I. M. | 2002 | 10.1086/339009 | Population |
| 131 | Mentrop L. | 2011 |  | Outcome |
| 132 | Moghaddam S. M. | 2019 | 10.3174/ajnr.A6159 | Outcome |
| 133 | Molyneux M. E. | 2000 | 10.1016/S0140-6736(99)00435-3 | Study design |
| 134 | Mpimbaza A. | 2007 |  | Outcome |
| 135 | Murphy S. | 1996 | 10.1016/s0035-9203(96)90260-6 | Outcome |
| 136 | Namazzi R. | 2017 |  | Outcome |
| 137 | Namazzi R. | 2019 |  | Outcome |
| 138 | Newton C. R. | 1997 | 10.1136/adc.76.3.219 | Outcome |
| 139 | Olumese P. E. | 1997 | 10.1093/tropej/43.4.217 | Outcome |
| 140 | Onifade E. U. | 2004 |  | Outcome |
| 141 | Osuntokun B. O. | 1987 | 10.1111/j.1600-0404.1987.tb07883.x | Exposure |
| 142 | Pasvol G. | 2005 | 10.1093/bmb/ldh059 | Outcome |
| 143 | Postels D. G. | 2013 | 10.1002/ana.24069 | Outcome |
| 144 | Postels D. G. | 2015 | [10.1002/ana.24477](https://dx.doi.org/10.1002/ana.24477) | Outcome |
| 145 | Postels D. | 2021 | 10.1002/ana.26177 | Outcome |
| 146 | Praygod G. | 2008 | 10.1186/1475-2875-7-210 | Study design |
| 147 | Raees M. | 2023 | 10.1097/01.ccm.0000907908.36162.4a | Outcome |
| 148 | Royo J. | 2019 | 10.1038/s41598-019-52579-7 | Outcome |
| 149 | Sahu P. K. | 2008 | 10.1179/136485908x300814 | Outcome |
| 150 | Savonius O. | 2023 | 10.1186/s12936-023-04556-9 | Outcome |
| 151 | Schemann J. | 2002 | 10.4269/ajtmh.2002.67.61 | Outcome |
| 152 | Schmutzhard J. | 2015 | 10.1186/s12916-015-0366-8 | Outcome |
| 153 | Senanayake N. | 1992 |  | Study design |
| 154 | Shabani E. | 2017 | 10.1111/pim.12438 | Duplicate |
| 155 | Shah D. | 2008 |  | Study design |
| 156 | Ssenkusu J. M. | 2016 | 10.1542/peds.2017-2709 | Outcome |
| 157 | Taylor T. E. | 1988 | 10.1056/NEJM198810203191602 | Outcome |
| 158 | Taylor T. E. | 1992 | 10.1111/j.1365-2249.1992.tb05851.x | Outcome |
| 159 | Thuma P. E. | 2011 | 10.1093/infdis/jiq041 | Outcome |
| 160 | Tinto H. | 2019 | 10.1016/s1473-3099(19)30300-7 | Outcome |
| 161 | Tripathy R. | 2007 | 10.1542/peds.2006-3171 | Outcome |
| 162 | Tu Z. | 2021 | 10.1038/s41598-021-94495-9 | Study design |
| 163 | Twomey P. | 2015 | 10.7326/m15-0910 | Outcome |
| 164 | Urtti S. | 2018 | 10.1093/ofid/ofy210.337 | Study design |
| 165 | van Hensbroek M. B. | 1996 | 10.1056/NEJM199607113350201 | Outcome |
| 166 | van Hensbroek M. B. | 1996 | 10.1093/infdis/174.5.1091 | Outcome |
| 167 | Warrell D. A. | 1982 | 10.1056/NEJM198202113060601 | Outcome |
| 168 | Crawley | 2000 | 10.1016/S0140-6736(99)07148-2 | Outcome |
| 169 | Akech A. | 2006 | 10.1371/journal.pctr.0010021 | Outcome |
| 170 | Conroy A. L. | 2019 | 10.4269/ajtmh.abstract2019 | Duplicate |
| 171 | Imam R. | 2025 | 10.1097/INF.0000000000004581 | Outcome |
| 172 | Chastang K. M. | 2023 | 10.4269/ajtmh.23-0022 | Duplicate |
| 173 | Jordan J. | 2024 | 10.3389/fneur.2024.1466941 | Outcome |
| 174 | Olowojesiku R. | 2024 | 10.1186/s12936-024-04843-z | Outcome |
